# Adaptation to pollination by fungus gnats underlies the evolution of pollination syndrome in the genus *Euonymus*

**DOI:** 10.1093/aob/mcad081

**Published:** 2023-08-23

**Authors:** Ko Mochizuki, Tomoko Okamoto, Kai-Hsiu Chen, Chun-Neng Wang, Matthew Evans, Andrea T Kramer, Atsushi Kawakita

**Affiliations:** Botanical Gardens, Graduate School of Science, The University of Tokyo, 3-7-1 Hakusan, Bunkyo-ku, Tokyo, Japan; Faculty of Applied Biological Sciences, Gifu University, Yanagido 1-1, Gifu, Japan; Department of Ecology and Evolution, Faculty of Biology and Medicine, University of Lausanne, Lausanne, Switzerland; Department of Life Science, Institute of Ecology and Evolutionary Biology, National Taiwan University, Taipei 10617, Taiwan; Chicago Botanic Garden, 1000 Lake Cook Road, Glencoe, IL 60022, USA; Chicago Botanic Garden, 1000 Lake Cook Road, Glencoe, IL 60022, USA; Botanical Gardens, Graduate School of Science, The University of Tokyo, 3-7-1 Hakusan, Bunkyo-ku, Tokyo, Japan

**Keywords:** Acetoin, Celastraceae, *Euonymus*, phylogeny, pollination by fungus gnat, pollination syndrome, pollinator shift, red flower

## Abstract

**Background and Aims:**

Dipteran insects are known pollinators of many angiosperms, but knowledge on how flies affect floral evolution is relatively scarce. Some plants pollinated by fungus gnats share a unique set of floral characters (dark red display, flat shape and short stamens), which differs from any known pollination syndromes. We tested whether this set of floral characters is a pollination syndrome associated with pollination by fungus gnats, using the genus *Euonymus* as a model.

**Methods:**

The pollinator and floral colour, morphology and scent profile were investigated for ten *Euonymus* species and *Tripterygium regelii* as an outgroup. The flower colour was evaluated using bee and fly colour vision models. The evolutionary association between fungus gnat pollination and each plant character was tested using a phylogenetically independent contrast. The ancestral state reconstruction was performed on flower colour, which is associated with fungus gnat pollination, to infer the evolution of pollination in the genus *Euonymus*.

**Key Results:**

The red-flowered *Euonymus* species were pollinated predominantly by fungus gnats, whereas the white-flowered species were pollinated by bees, beetles and brachyceran flies. The colour vision analysis suggested that red and white flowers are perceived as different colours by both bees and flies. The floral scents of the fungus gnat-pollinated species were characterized by acetoin, which made up >90 % of the total scent in three species. Phylogenetically independent contrast showed that the evolution of fungus gnat pollination is associated with acquisition of red flowers, short stamens and acetoin emission.

**Conclusions:**

Our results suggest that the observed combination of floral characters is a pollination syndrome associated with the parallel evolution of pollination by fungus gnats. Although the role of the red floral display and acetoin in pollinator attraction remains to be elucidated, our finding underscores the importance of fungus gnats as potential contributors to floral diversification.

## INTRODUCTION

The observation that distantly related plants sharing the same pollinators exhibit a set of similar floral characteristics has led to the idea of the pollination syndrome, suggesting that the shared floral traits are the result of convergent adaptation towards particular pollinators (Delpino, 1873–1874; Vogel, 1954; van der Pijl, 1960; Fenster *et al.*, 2004; Ashworth *et al.*, 2015; Johnson and Wester, 2017). Pollinating vectors include various animal groups that differ in morphology, behaviour and sensory properties. Traditionally, distinct pollination syndromes have been proposed for pollination by bees, birds, bats, (hawk)moths, non-flying mammals, butterflies, beetles, flies, carrion flies, long-tongued flies and wasps (Fægri and van der Pijl, 1979; Proctor *et al.*, 1996; Willmer, 2011). Because this categorization assumes that each set of floral traits is shaped by pollinator-mediated selection, a phylogenetic test on the evolutionary association between pollinator types and floral traits provides support for the pollination syndrome hypothesis (Ollerton *et al.*, 2009). However, evidence is largely limited to systems involving large pollinators, such as bees, birds and bats (i.e. Whittall and Hodges, 2007; Smith *et al.*, 2008; Tripp and Manos, 2008; Martén-Rodríguez *et al.*, 2009; Lagomarsino *et al.*, 2017). Comparatively, little effort has been made in the analysis of floral traits in other pollination systems, especially those involving small insects and small flowers (Dellinger, 2020).

The true flies, Diptera, are among those insects whose evolutionary relationship with floral traits remains unexplored. Dipteran insects are known to transfer the pollen of a significant fraction of plant species, particularly in alpine areas (Lefebvre *et al.*, 2014; Orford *et al.*, 2015) and agricultural environments (Warren *et al.*, 1988; Elberling and Olesen, 1999; Ssymank *et al.*, 2008), and act as the fundamental pollinators of certain plant lineages, such as Araceae (Chartier *et al.*, 2014). Pollination by dipteran insects, or myiophily (myophily), has often been grouped into three types (Fægri and van der Pijl, 1979; Rosas-Guerrero *et al.*, 2014; Ashworth *et al.*, 2015). One is the generalized pollination system in which a single plant is pollinated by flies of diverse families, such as blow flies and hoverflies. The plants normally have yellow or white, bowl- to open-shaped and nectar-producing flowers (Zych *et al.*, 2014; Garcia *et al.*, 2022), which are sometimes specifically called myiophilous flowers (Willmer, 2011). Second is sapromyiophily, in which the plants are pollinated by a fly guild including house flies, dung flies and blow flies that use animal dung and/or carrion for feeding and reproduction. The plants possess dull, dark-purplish flowers emitting floral scent that resembles animal dung or carrion (Chen *et al.*, 2015). Lastly, plants pollinated by long-tongued Nemestrinidae flies share purple- to cerise-coloured, narrow-tubed flowers (Manning and Goldblatt, 1996), although the geographical occurrence is restricted to southern Africa.

Recent meta-analyses of pollination syndrome show that the floral traits of these Diptera-pollinated plants are good predictors of the associated pollinators (Rosas-Guerrero *et al.*, 2014; Ashworth *et al.*, 2015). Nevertheless, such classical categorization has often been questioned. This is because pollination by Diptera involves >70 fly families (Larson *et al.*, 2001), and they are diverse in both adult and larval habitats, collectively utilizing the broadest range of insect niches (Marshall, 2012). Consequently, the selective pressure imposed on plants might be much more diverse than currently categorized (Larson *et al.*, 2001; Inouye *et al.*, 2015). For example, the genera *Ceropegia* (Apocynaceae) and *Aristolochia* (Aristolochiaceae) independently evolved kleptomyiophily, in which the plants lure kleptoparasitic flies of Milichiidae and Chloropidae using an odour mimicking freshly killed insect bodies (Oelschlägel *et al.*, 2014; Heiduk *et al.*, 2016). Likewise, some fly-pollinated plants often exploit female flies by mimicking oviposition sites (Stökl *et al.*, 2011). As such, the floral adaptations in plants pollinated by different groups of dipteran insects are likely to differ from one another (Johnson and Schiestl, 2016), but we lack evidence on whether different dipteran insect groups have driven the evolution of distinct floral characteristics.

Plants pollinated by fungus gnats are putative examples exhibiting convergence in floral traits (Okuyama *et al.*, 2008; Mochizuki and Kawakita, 2018). Fungus gnats are small dipteran insects belonging to several families in the superfamily Scialoidea in the suborder Nematocera, such as Mycetophilidae and Sciaridae. Many species inhabit forests, where their larvae feed on fungal materials, decomposing plant matter or bryophytes (Jakovlev, 2011; Okuyama *et al.*, 2018). Pollination by fungus gnats has generally received little attention but is now known in 12 plant families (Mochizuki and Kawakita, 2018; Guo *et al.*, 2019). Examples include trap pollination in the genus *Arisaema* (Vogel and Martens, 2000; Matsumoto *et al.*, 2021) or *Pterostylis* orchids that exploit copulatory behaviour of male fungus gnats (Reiter *et al.*, 2019). Yet, many fungus gnat-pollinated plants offer nectar as a reward (Okuyama *et al.*, 2004). In our previous study, we found that seven plant species belonging to five families in Japan are pollinated predominantly by fungus gnats and reward them with nectar (Mochizuki and Kawakita, 2018). The flowers of the seven species and the previously known fungus gnat-pollinated *Mitella* are strikingly similar to each other, in that they share a dark red floral display, short stamens, flat-shaped flowers and exposed nectaries. The observation that distantly related plants that share fungus gnat pollinators possess a set of similar floral characters fits the concept of a pollination syndrome (Fenster *et al.*, 2004).

In this study, we tested whether adaptation to fungus gnat pollination has driven the evolution of convergent floral characters in the spindle tree genus *Euonymus* (Celastraceae). This test requires information on the phylogenetic relationship and pollinator data of closely related plant species with variable floral characters (e.g. Whittall and Hodges, 2007; Okuyama *et al.*, 2008; Smith *et al.*, 2008; Lagomarsino *et al.*, 2017). The genus *Euonymus* contains 129 species of shrubs or shrublets distributed mainly in the Northern Hemisphere, with a centre of diversity in East Asia (Ma, 2001). The flowers of *Euonymus* species are typically pale green or yellowish white (henceforth ‘white’), but roughly one-quarter of the species have flowers with dull red or dark red colour (henceforth ‘red’) (Ma, 2001). The known fungus gnat-pollinated *Euonymus lanceolatus*, *E. melananthus* and *E. tricarpus* possess dark red flowers and occur in dark forest floor habitats or along forest streams, which are common habitats of fungus gnat-pollinated plants (Mochizuki and Kawakita, 2018). The overall floral morphology is similar among species, in that flowers are open and flat in shape, with an exposed nectary disc. In contrast, filament length varies among species, ranging from almost sessile to as long as the petal length (Ma, 2001). Floral scent is normally detectable to the human nose and is also variable among species, with fungus gnat-pollinated species typically having a distinct fermented scent (K. Mochizuki, pers. obs.). Such variations in flower colour, stamen length and floral scent within the genus allow us to test the hypotheses of floral evolution associated with pollination systems.

We investigated the pollinators and floral characters (colour, stamen length and floral scent) of five red-flowered *Euonymus* species distributed in East Asia and North America (pollinator information of three species is derived from the paper by Mochizuki and Kawakita, 2018), five white-flowered *Euonymus* species in Japan and *Tripterygium regelii*, which served as an outgroup. The flower colour was evaluated using insect colour vision models to account for the perception of flower colour by insect vision (Chittka, 1992; Troje, 1993). The obtained pollinator data and floral characters were mapped to a molecular phylogeny to test the evolutionary association between fungus gnat pollination and each plant trait. We then reconstructed the ancestral state of flower colour, which is strongly associated with fungus gnat pollination, to infer the evolutionary scenario of fungus gnat pollination in the genus *Euonymus*.

## MATERIALS AND METHODS

### Observations of pollinators

**Fig. 1.**
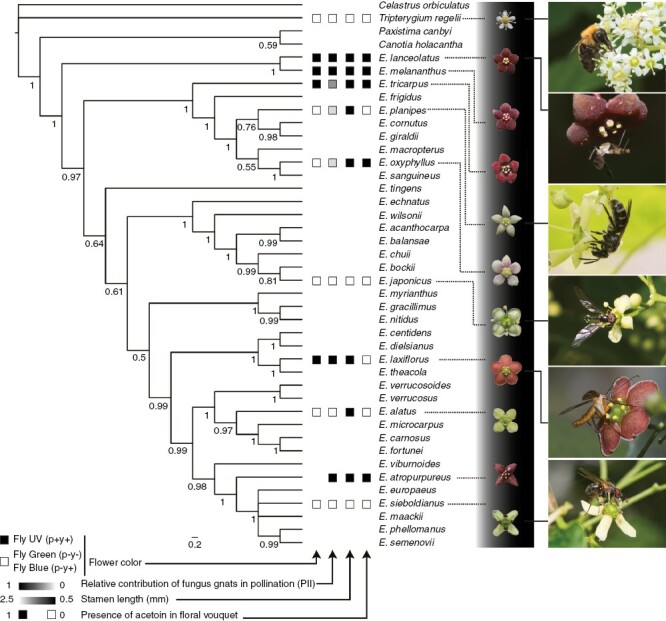
Phylogenetic relationship of 38 *Euonymus* species with outgroups, based on sequences of two nuclear (ITS and ETS) regions, with images of flowers and selected floral visitors of the studied plants. Numbers beside the nodes indicate Bayesian inference posterior probability (percentage) support. The four squares beside the tip of the branches indicate, from left to right, flower colour, contribution of fungus gnats relative to the whole pollination, stamen length and the presence of acetoin in floral scent. For flower colour, the species with fly-UV (p+y+) are red-flowered species, and the species with fly-Green (p−y−) and Fly-Blue (p−y+) are white-flowered species (see Results and Fig. 2).

To explore the relationship between floral traits and pollinators, we investigated the pollinators of plant species with different morphology and/or colour relative to species pollinated by fungus gnats. In Japan, 18 *Euonymus* species are native: 14 have white or yellowish flowers and four possess dark red flowers (Fig. 1). For the species with white flowers, we studied the pollinator assemblages and floral characters of *Euonymus alatus*, *E. oxyphyllus* and *E. planipes* with short stamens, and *E. japonicus* and *E. sieboldianus* with long stamens. As the outgroup reference, *T. regelii*, which is distributed in the cool-temperate area of Japan and possesses white flowers with long stamens, was studied. Furthermore, we conducted observations on red-flowered species distributed outside Japan: *E. laxiflorus* distributed in Taiwan and East Asian countries and *E. atropurpureus* endemic to North America. The field observations were performed during both daytime (0900–1800 h) and night-time (1800–2200 h) during 2015–2019. Each species was observed in one to three populations (for the details of field observations, see Supplementary Data Table S1). The insect visitors were captured whenever possible and identified to genus level for fungus gnats and family level for other arthropods. We assigned sciarid flies as Sciaridae spp. owing to the difficulty of distinguishing genera and species. For the specimens collected from *E. lanceolatus*, *E. melananthus* and *E. tricarpus*, which were used for the previous study (Mochizuki and Kawakita, 2018), identification was performed again with the specimens collected in this study. For family-level identification of Diptera, we followed Marshall (2012). The pollinator importance index (PII) was calculated for each functional pollinator group following the method described by Mochizuki and Kawakita (2018). Data on flower visitors for multiple populations were merged because geographical variation was not very apparent. For the functional pollinator group, ‘Bee’ and ‘Other long-proboscid arthropods’ were added here. Note that the field observations of *E. planipes* and *E. tricarpus* were conducted in a co-flowering population on Rishiri Island located at the northernmost part of Hokkaido, Japan. Although there is a possibility of over- or under-estimation of the PII value for some shared visitors, we consider that the overall conclusion on the most important pollinator is largely unaffected, because such visitors were not the most frequent.

### Analysis of flower colour using an insect colour vision model

Spectral reflectance across the 300–700 nm range was determined using an Ocean Optics USB4000 spectrometer (Ocean Insight, Orlando, FL, USA) and a reflection probe (R-600-7-SR-125F) held at 45° to the petal surface. As a light source we used a DH-2000-BAL deuterium tungsten halogen light (Ocean Insight) with a ~200–2000 nm spectral range. An Ocean Optics WS-1 diffuse reflectance standard (Ocean Insight) was used for calibration. For each individual, measurements were made for the petals of five flowers, and the average reflectance was obtained. Replicates were taken for five to nine individuals for each species, except for *E. planipes*, in which data were obtained from one individual. Data for *E. atropurpureus* were not available. The raw floral reflectance spectra were processed to generate data at 1 nm intervals and visualized using the package *pavo* (Maia *et al.*, 2019) in R v.3.3.3 (R Core Team, 2017).

Flower colour is the primary cue to attract pollinators, but the flowers might be perceived differently by human vision and that of pollinating animals because the visual systems are different among animals (Chittka and Menzel, 1992; Chittka *et al.*, 1994; Osorio and Vorobyev, 2008; Lunau *et al.*, 2011; Bischoff *et al.*, 2013; Shrestha *et al.*, 2019). Therefore, we investigated the flower colours seen through the insect eyes using the spectral reflectance data from the above analysis and the colour vision model for bees (Chittka, 1992) and flies (Troje, 1993). Bees have trichromatic vision, with receptors most sensitive to ultraviolet (UV), blue and green (Chittka, 1992). Dipteran insects are known to recognize colours in four categories using two types of ommatidia, the p- and y-type receptors, which are composed of pairs of retinula cells R7p and R8p, and R7y and R8y, respectively (Troje, 1993; Lunau, 2014). The ommatidium produces either plus or minus signal based on the relative excitation of the two retinula cells. Consequently, four combinations of signals from the two types of ommatidia (p+y+, p+y−, p−y+ and p−y−) allow the assignment of four colour categories. This visual system seems to be conservative in the suborder Brachycera, because *Musca domestica* (Hardie and Kirschfeld, 1983), *Calliphora erythrocephala* (Hardie, 1985), *Eristalis tenax* (Bishop, 1974; Horridge *et al.*, 1975) and *Drosophila melanogaster* (Yamaguchi *et al.*, 2010) share the same system. The detailed visual systems of the suborder Nematocera have never been investigated. Thus, we instead used the colour vision model of the blowfly *Lucilia* (Troje, 1993). Analysis was performed using the package *pavo* (Maia *et al.*, 2019), and the spectral reflectance of one leaf for each species was measured and used for calculation of colour loci.

### Floral scent analysis

The chemical composition of floral scent was investigated for the plant species whose pollinators were investigated. The collection of floral scent was performed using solid phase microextraction (SPME; Supelco, 50 μm/30 μm DVB/CAR/PDMS; Sigma-Aldrich, Inc., Saint Louis, MO, USA) for most of the species and Tenax-TA (60 mg; mesh 80–100; GL Sciences, Tokyo, Japan) for additional samples of *E. oxyphyllus*, *E. lanceolatus* and *E. melananthus*. For *E. atropupureus*, sampling was performed only by Tenax.

The collection was performed using potted trees or plants growing in the populations where pollinator observations were conducted during the time when the pollinator activity was high for each plant species. One to nine individuals were used for sampling.

Before sampling using SPME, one to ten flowers were enclosed in a 15 or 30 mL glass vial, and the vial’s neck was covered using aluminium foil. Subsequently, the fibre assemblies were exposed to floral headspace for 15–60 min. The flowers used for each sampling originated from a single individual, and the number of flowers and sampling duration were adjusted depending on the strength of floral scent to the human nose and the quality of the obtained data. For the control, the same number of pedicels as the number of flowers in the floral sample was cut and placed inside another vial. Every vial was washed with 2 mL of acetone and 6 mL of hexane and dried before the experiments.

For volatile collection using Tenax-TA, the headspace air of a single intact inflorescence or leaf-bearing branch enclosed with a polyester oven bag was collected. We allowed the air, after cleaning by activated charcoal, to go through the Tenax-TA-filled glass tube inserted into the bag with an air pump (MP-2N; SIBATA, Tokyo, Japan) at a rate of 100 mL min^−1^ for 2–3 h. The glass tubes containing captured floral volatiles were stored at −30 °C until analysis. The volatiles captured by Tenax-TA were eluted from the adsorbent with 2 mL of acetone. The liquid was then concentrated to 10 μL with a N_2_ flow, and an aliquot (1 μL) of each sample was used for the analysis with 10 ng of eicosane as an internal standard.

Scent samples were analysed using gas chromatography–mass spectrometry (GC-MS) of GC-17A coupled with QP5050A, GCMS-QP2010 or GCMS-QP2010 SE systems (Shimadzu, Kyoto, Japan). For GC, we used a DB-5 capillary non-polar column (30 m × 0.25 mm; film thickness, 0.25 µm; Agilent Technologies, CA, USA) or Rtx-5SilMS capillary column (30 m × 0.25 mm; film thickness, 0.25 μm; Restek, Bellefonte, PA, USA). The injector was set to the splitless mode at 250 °C for 1 min. The oven temperature was programmed at 40 °C for 5 min, followed by an increase by 8 °C min^−1^ to 280 °C, where it was held for 10 min. For samples of *E. laxiflorus*, the program was set at 40 °C for 5 min, followed by an increase by 5 °C min^−1^ to 200 °C and 10 °C min^−1^ to 280 °C, where it was held for 5 min. We also ran n-alkane ladders (C_7_ to C_33_) for each piece of equipment, column and oven temperature program, to be used for calculation of the retention index (RI) for each compound in floral scent samples.

For identification of the compounds, we compared the fragments of each floral volatile peak with those of the references contained in the NIST 05s or 21 spectral mass library, and the RI was calculated based on the retention time of each compound and the authentic standards composed of n-alkanes and compared with those reported in NIST Chemical WebBook (https://webbook.nist.gov/chemistry/). For the compound acetoin, which was revealed to characterize the scent of fungus gnat-pollinated plants, identification was performed by comparing the GC retention time and mass spectra of the samples with those of an authentic standard (CAS No. 513-86-0; Tokyo Chemical Industry, Tokyo, Japan).

By comparing the volatiles emitted from flowers and controls, volatiles emitted only from flowers were considered as floral volatiles. In contrast to Tenax-TA, the SPME fibres have higher sensitivity but have a bias in the collection of compounds, thus the peak mass area does not reflect the true quantity of the compounds (Povolo and Contarini, 2003). SPME captured more chemicals that are detected in trace amounts by the Tenax method, such as sesquiterpenes (see Results). Nonetheless, we used the data generated from both collection methods because the major compound was not different between the sampling method, and the subsequent analysis gave the same conclusion even when Tenax samples were omitted (see Results). Similarities of compositions were visualized by non-parametric multidimensional scaling (NMDS) analysis, using the *metaMDS* function embedded in the package *vegan* (Oksanen *et al.*, 2018) in R v.3.3.3 (R Core Team, 2017). The floral scent compositions of white- and red-flowered species were compared by permutational multivariate analysis of variance (PERMANOVA; Anderson, 2017) based on Bray–Curtis dissimilarities, using the *adonis2* function. The compositions of *E. laxiflorus* were compared with other red-flowered species and white-flowered species based on Tukey’s honest significant differences between groups, using the function *TukeyHSD* and *betadisper*, because *E. laxiflorus* did not share the primary compound with other red-flowered species. We then investigated the contribution of each chemical compound to the difference of floral scent composition between red- and white-flowered species using the *simper* function in the package *vegan* (Oksanen *et al.*, 2018).

### Phylogenetic reconstruction

For the purpose of phylogenetic analysis, we downloaded DNA sequence data on regions used by Simmons *et al.* (2012) and Li *et al.* (2014) for 32 *Euonymus* species and outgroups including *T. regelii* from NCBI (https://www.ncbi.nlm.nih.gov/). We sampled the leaves and extracted DNA of species that were not included in the previous reports (*E. atropurpureus*, *E. japonicus*, *E. lanceolatus*, *E. melananthus*, *E. planipes* and *E. tricarpus*), following a modified cetyltrimethylammonium bromide protocol (Okuyama and Kawakita, 2012). The voucher specimens were deposited in the Herbarium of the University of Tokyo (TI), and the accession numbers are provided in the Supplementary Data (Table S2).

Two nuclear regions (ITS and ETS) and three chloroplast DNA intergenic spacers (*rp136-infA-rps8*, *trnC-ycf6* and *psbA-trnH*) were used in the previous papers. However, a preliminary analysis using the incongruence length difference test (Farris *et al.*, 1994), as implemented in the partition homogeneity test in PAUP (Swofford, 2003), suggested a significant incongruence between nuclear and chloroplast genes (*P* = 0.04). Furthermore, the phylogeny created using chloroplast genes yielded a tree with very low resolution, hence we decided to use nuclear regions for phylogenetic reconstruction.

ITS and ETS regions were sequenced on an ABI 3130 Genetic Analyzer (Applied Biosystems, Foster City, CA, USA). We used the primers described by Li *et al.* (2014). Obvious sequence errors were corrected manually using MEGA v.7 (Kumar *et al.*, 2016) and the obtained sequences were aligned using MAFFT v.6.901 (Katoh and Toh, 2008) under the default settings. The obtained sequences were deposited in NCBI, and the GenBank accession numbers are provided in the Supplementary Data (Table S2).

Phylogenetic reconstruction was performed with the Bayesian inference method, using MrBayes (Ronquist and Huelsenbeck, 2003), following the method described by Ito *et al.* (2017). The GTR+G model was selected as the most appropriate evolutionary model based on Bayesian inference criterion implemented in Kakusan4 (Tanabe, 2011). Based on the model selected, we performed two separate runs of Metropolis-coupled Markov chain Monte Carlo (MCMCMC) analyses, each with a random starting tree and four chains (one cold and three hot). The MCMCMC was 10 million generations long, and the chain was sampled every 1000 generations from the cold chain. After checking that the value of the average s.d. of split frequency was <0.01, the first 2500 sample trees (25 % of 10 000 sample trees) were discarded as burn-in. The 50 % majority-rule consensus tree of all post-burn-in trees was generated using FigTree v.1.3.1 (Rambaut, 2009).

### Phylogenetic correlation among pollinator type and floral characters

Correlated evolution of pollination type and three floral characters (flower colour, flower morphology and the floral scent) was tested using independent contrasts, as implemented in the PDAP module (Midford *et al.*, 2010) of Mesquite v.3.2 (Maddison and Maddison, 2017). The phylogeny reconstructed using nuclear regions was used for the following analysis. Pollinator types were coded as continuous data using the PII of fungus gnats. The flower colours were coded as 1 or 0 for fly-UV (p+y+) and others, including fly-Blue (p−y+) and fly-Green (p−y−), respectively, that correspond to red and white flowers seen in human vision (see Results). Given that short stamens are suggested to be important in effective pollen deposition in some fungus gnat-pollination systems (Mochizuki and Kawakita, 2018), the average length of the stamen (filament + anther) was also subjected to the analysis as a morphological character.

The flowers were collected in the field and preserved in 70 % ethanol. The flowers were photographed under a stereomicroscope with a scale, and the stamen length was measured using the software ImageJ v.1.48 (Schneider *et al.*, 2012). For each species, measurement was conducted on five to seven individuals, and two to five flower replicates were taken for each individual. For the floral scent data, we focused on the compound acetoin, because the scent analysis revealed the dominance of acetoin in four fungus gnat-pollinated species, which was mostly absent in non-fungus gnat-pollinated species. The presence or absence of acetoin in floral scent was coded as 1 or 0 for each species because of the uncertainty of quantification by the SPME method.

### Ancestral state reconstruction

Given that the phylogenetically independent contrast suggested a strong association between red flower colour and pollination by fungus gnat, the transition of flower colour should provide information on the transition of pollination system and floral traits in the genus *Euonymus* and related genera. Therefore, we used flower colour as a proxy for a pollination system and investigated how the flower colour has shifted by reconstructing the ancestral state of the flower colour on the phylogeny reconstructed above.

The flower colour of species included in the phylogeny was categorized as either red or white, based on the description by Ma (2001), because the colour vision analysis suggested that dipteran pollinators are likely to discriminate between red and white flowers. The species whose floral colour is referred to as chocolate, dark purple, dark red, purplish or red(-ish) were assigned to red (1), whereas species whose floral colour is described as white(-ish), yellow(-ish), green(-ish) or cream were assigned as white (0). Some species, such as *E. oxyphyllus*, *E. bockii* and *E. carnosus*, have flowers with red-coloured veins on white petals, but their colour was assigned as white because the overall display is not red.

The analyses were conducted using a parsimony and a maximum likelihood approach using Mesquite v.3.2 (Maddison and Maddison, 2017) and a reversible jump Markov chain Monte Carlo (RJMCMC) approach using the MultiState module as implemented in BayesTraits v.3.0.2 (Pagel and Meade, 2006). Each RJMCMC analysis was run for 1 000 000 generations, and parameters and ancestral states were sampled every 1000 generations with an exponential hyper-prior, with a mean on a uniform interval from 0 to 100. The first 100 000 iterations were discarded as burn-in. The reconstructed ancestral states were plotted onto the nuclear gene tree derived from MrBayes. We then compared the likelihood of the model between ancestral states by setting the focal nodes to take an alternative character using the *fossil* command. To access the evidence level, Bayes factors were calculated as 2 × [log(marginal likelihood of model 1) − log(marginal likelihood of model 2)]. Bayes factors higher than two indicate positive evidence, and Bayes factors higher than five indicate strong evidence of support for the best model (Pagel *et al.*, 2004).

## RESULTS

### *Pollinators of* Euonymus *species*

The pollination systems of many studied species involved many insect taxa, with particular functional pollinator groups making major contributions to pollination, and there was a clear-cut difference in pollinators between red- and white-flowered species (Table 1; Fig. 1). In red-flowered *E. atropurpureus*, ten individuals of two species of the *Neoempheria* (Mycetophilidae) and four uncaptured and unidentified mycetophilid fungus gnats were observed during the 19 h observation during the daytime. In *Euonymus laxiflorus*, a total of 115 individuals of 47 species of 24 insect families from seven pollinator groups were observed during the 36 h observation. Around dusk, the fungus gnats *Proceroplatus* sp. (Keroplatidae), *Neoempheria* spp. (Mycetophilidae) and Sciaridae spp. were observed frequently visiting flowers. Both *E*. *atropurpureus* and *E. laxiflorus* depend on fungus gnats for pollination (PII = 1.00 and 0.958, respectively). Fungus gnats were observed contacting anthers with their coxa when they fed on nectar secreted from the nectary disc surrounding the base of the pistil and stamens (Supplementary Data Fig. S1). White-flowered species were not pollinated by fungus gnats but predominantly by bees, brachyceran flies or coleopteran insects (Table 1). In comparison to red-flowered species, pollen-feeding insects, such as bees, hoverflies and coleopteran insects, were more frequently observed during the daytime, whereas nocturnal visitors were scarce. In total, 684 individuals of 209 species belonging to 59 families were recorded (Supplementary Data Table S3). Visitors were observed to contact anthers and stigmas when they fed on pollen or nectar. In species with longer stamens, pollen grains were carried on the lateral thorax or the entire body of the pollinators, whereas in species with sessile stamens, pollen grains were carried on the head and the ventral sides of the thorax and abdomen of pollinators. Among long-stamened species, the primary and secondary pollinators were different among species: beetles and bees in *E. japonicus*; hoverfly and non-syrphid Brachycera in *E. sieboldianus*; and bees and hoverflies in *T. regelii*, respectively (Table 1). In short-stamened species, the primary and secondary pollinators were bees and other Hymenoptera in *E. alatus*, beetles and fungus gnats in *E. oxyphyllus*, and bees and other Nematoceran flies in *E. planipes*, respectively (Table 1). Within each functional group, the pollinator taxa were different; the bee pollinators were small-sized halictid and apid species in *E. alatus*, small-sized andrenid and halictid bees in *E. planipes*, and bumblebees in *T. regelii*; the coleopteran insects were small-sized Cerambycidae in *E. oxyphyllus* and large-sized Scarabaeidae in *E. japonicus* (Supplementary Data Table S3).

**Table 1.** Importance of each functional pollinator group to the *Euonymus* species and *Tripterygium regelii*

Functional group	Red-flowered species					White-flowered species					
	*E. atropurpureus*	*E. lanceolatus*	*E. laxiflorus*	*E. melananthus*	*E. tricarpus*	*E. alatus*	*E. japonicus*	*E. oxyphyllus*	*E. planipes*	*E. sieboldianus*	*T. regelii*
Fungus gnat	**1.00**	**0.867**	**0.958**	**0.918**	**0.551**	–	0.003	0.199	0.179	–	–
Crane fly	–	–	0.002	–	0.070	0.012	–	–	0.001	–	–
Other Nematocera	–	–	0.001	–	0.148	–	–	–	0.276	–	–
Hoverfly	–	–	–	–	–	0.033	0.009	–	0.005	**0.646**	0.127
Non-syrphid Brachycera	–	0.115	0.039	0.005	0.034	0.059	0.076	0.041	0.161	0.345	–
Coleoptera	–	–	–	0.034	0.063	0.154	**0.763**	**0.731**	0.051	0.008	–
Ant	–	0.015	–	–	0.003	0.012	0.003	–	–	–	–
Bee	–	–	0	–	–	**0.521**	0.119	–	**0.291**	–	**0.718**
Other Hymenoptera	–	–	–	–	0.131	0.208	0.025	0.029	0.036	–	0.154
Lepidoptera	–	–	–	–	–	–	–	–	–	0.001	–
Other long-proboscid arthropods	–	0	–	–	–	–	–	–	–	–	–
Other short-proboscid arthropods	–	0.003	–	0.043	–	–	–	–	–	–	–

Note that pollinator importance index values for *E. lanceolatus*, *E. melananthus* and *E. tricarpus* were calculated based on specimens collected in an earlier study (Mochizuki and Kawakita, 2018). The group contributing the most to each plant species is highlighted in bold. A dash in the column indicates the absence of flower visitation.

### Spectral reflectance and colour vision analysis

**Fig. 2.**
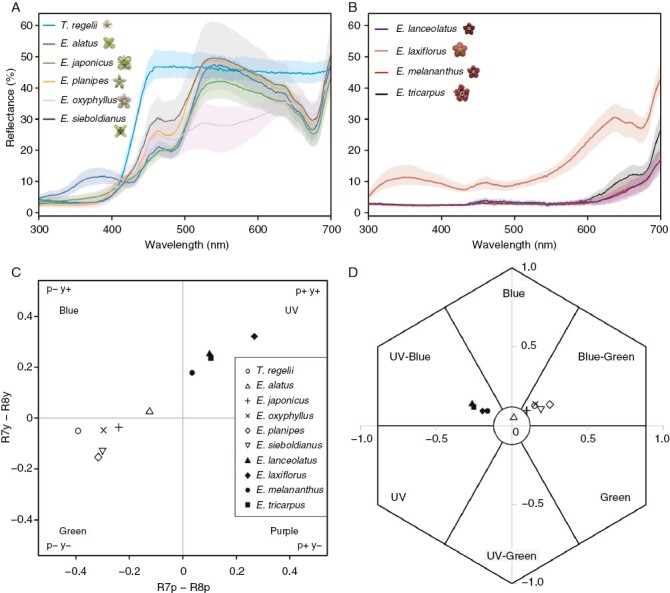
Spectral reflectance and insect perception of petal colour. Spectral reflectance of the petal of the white-flowered *Euonymus* species (A) and red-flowered *Euonymus* species (B). Results of colour vision analysis using fly (C) and bee (D) colour vision. in In D, the circle at the origin has a radius of 0.1 hexagon units, and samples within the circle are indistinguishable from the background by bee colour vision. The symbols in D represent the same species as in C.

The white flowers generally had higher reflection than red flowers, with many species having reflection patterns with peaks at ~550 nm (Fig. 2A). In comparison to the white flowers, the red flowers mostly absorbed the light and reflected light at wavelengths of >650 nm, where flies and bees are not sensitive (Fig. 2B). The flowers of *E. laxiflorus*, which look brighter to human eyes than other red flowers, reflected UV with a peak at ~350 nm. Red flowers fell into the p+y+ region (fly-UV) in fly colour vision and into the UV–blue region in bee colour vision, whereas white flowers generally fell into the p−y− region (fly-Green) in fly colour vision and into the blue–green region in bee colour vision (Fig. 2C, D); thereby, the red flowers and white flowers were perceived as different colours both by flies and bees.

### Floral scent

A total of 78 compounds were detected from the 39 samples of ten *Euonymus* species and *T. regelii* (Supplementary Data Table S4). In the red-flowered *E. atropurpureus*, *E. lanceolatus*, *E. melananthus* and *E. tricarpus*, acetoin (3-hydroxy-2-butanone) was always observed and accounted for 29–99 % of the total peak area of compounds within individual samples. There was little overlap in compounds between species except for acetoin, but the ketones 2-heptanone, 2-nonanone and 2-undecanone were repeatedly detected in *E. lanceolatus* and *E. melananthus*, and some sesquiterpenes, such as copaene, β-caryophyllene, germacrene D and δ-cadinene, were repeatedly observed in *E. lanceolatus*, *E. melananthus* and *E. tricarpus*. The floral scent of *E. laxiflorus* did not contain acetoin; instead, 2-heptanone, 2-heptanol, 1-mthoxynaphthalene and one unidentified aliphatic (*m*/*z* = 265, 41, 84, 55, 68, 97, 161) were consistently observed across the samples. In the white-flowered species, monoterpenes, such as α-pinene, sabinene, m-cymene and limonene, were the major compounds in *E. japonicus*, *E. planipes*, *E. sieboldianus* and *T. regelii*. In *E. alatus* and *E. oxyphyllus*, sesquiterpenes, including β-caryophyllene and α-farnesene, were the major compounds. Acetoin was almost unique to red-flowered species, except for *E. oxyphyllus*, in which a small amount of acetoin was detected.

**Fig. 3.**
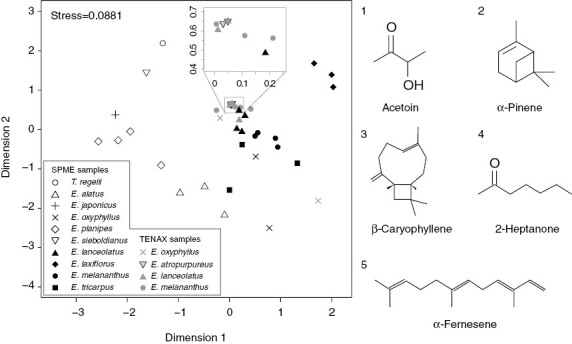
Non-metric multidimensional scaling (NMDS) based on Bray–Curtis dissimilarities of the scent composition (78 compounds) of ten *Euonymus* species and *Tripterygium regelii*. The structures of the top five compounds contributing to the 49.8 % of overall difference in floral scent among species as revealed by SIMPER analysis are shown beside the NMDS plot.

The floral scent composition was significantly different between red- and white- flowered species (PERMANOVA, *F* = 8.75, *R*^2^ = 0.19, *P* = 0.001). Pairwise comparison found no difference between the floral scents of *E. laxiflorus* and other red-flowered species (adjusted *P* = 0.458), whereas the scents of white-flowered species differed from those of *E. laxiflorus* (adjusted *P* = 0.004) and other red-flowered species (adjusted *P* < 0.001). Acetoin was the primary explanatory compound (SIMPER contribution 27.2 %) to the difference in floral scent between white- and red-flowered species, followed by α-pinene, β-caryophyllene, 2-heptanone and α-farnesene, which composed 49.8 % of the total explanatory variables (Fig. 3). When Tenax samples were omitted from the above analysis, the SIMPER analysis became slightly different: the contribution of acetoin dropped to 14.8 %, and the fifth compound become m-cymene; however, the overall results of other statistics, including NMDS and PERMANOVA, were not changed (Supplementary Data Fig. S2).

### Phylogenetic correlation between pollinator types and floral traits and ancestral character reconstruction

The red-flowered *E. lanceolatus* and *E. melananthus*, both endemic to Japan, were recovered as the basal-most lineage of the genus *Euonymus* (Fig. 1). The studied, red-flowered species did not form a clade and instead were distributed in multiple lineages across the genus (Fig. 1). Phylogenetically independent contrasts showed that the PII of fungus gnats, red flower colour and the presence of acetoin in floral scent were mostly exhibited positively correlations with each other, whereas stamen length was negatively correlated with PII of fungus gnats and other floral characters (Table 2). The association between colour and acetoin emission was not statistically supported.

**Table 2.** Result of phylogenetically independent contrast

Pairwise contrasts of floral characters	Pearson’s correlation	*P*-value
PII of fungus gnats vs. red flower colour	0.870	<0.001
PII of fungus gnats vs. stamen length	−0.787	0.002
PII of fungus gnats vs. acetoin presence in floral scent	0.714	0.01
Red flower colour vs. stamen length	−0.579	0.04
Red flower colour vs. acetoin presence in floral scent	0.536	0.11
Stamen length vs. acetoin presence in floral scent	−0.678	0.02

**Fig. 4.**
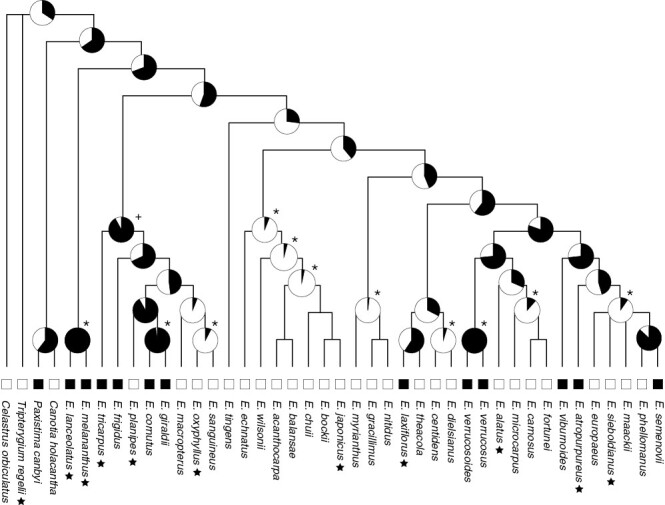
The result of ancestral reconstruction on flower colour based on Bayesian inference. The symbols ‘+’ and ‘*’ indicate that the Bayes factors are >2 and >5, respectively. The species at which pollinators were investigated are indicated with stars. Although the statistical supports on the basal branches are not strong, the transitions of flower colour between red and white are bidirectional within the genus.

The evolution of the red flower seemed to have multiple origins within the genus *Euonymus* (Fig. 4), although the reconstruction of ancestral states did not have strong support, except for several nodes close to the tip of the tree. The red flower was likely to have been acquired in the common ancestor of the genus *Euonymus* or that of the genera *Euonymus*, *Paxistima* and *Canotia*. The likely colour shifts supported by the Bayes factor were observed from red to white at the ancestor of *E. oxyphyllus* and *E. sanguiaenus* and from white to red in *E. semenovii*. Thus, the shift of flower colour might be bidirectional between red and white. Given that several clades formed by white-flowered species nested within the lineage containing red-flowered species, flower colour might have shifted from red to white at the ancestor located at the node from the basal-most node of *Euonymus*. Consequently, the red flower colour might have evolved independently in *E. laxiflorus* and *E. atropurpureus* from *E. tricarpus*, *E. melananthus* and *E. lanceolatus*. The overall result was not notably different between the Bayesian, maximum likelihood and parsimony reconstruction methods (Supplementary Data Fig. S3).

## DISCUSSION

The observation that red-flowered *Euonymus* species, including Taiwanese *E. laxiflorus* and North American *E. atropurpureus*, were pollinated by fungus gnats, whereas white-flowered species were not, confirmed the predictive power of red floral colour for fungus gnat pollination in the genus *Euonymus* (Table 1). The red- and white-flowered species were different in floral display as perceived by flies and bees and in floral scent compositions (Figs 2 and 3). Importantly, using phylogenetically independent contrasts, we detected a significant association of red floral display, short stamen and acetoin emission to pollination by fungus gnats (Table 2). Ancestral reconstruction suggested that red flowers might not have a single origin; thus, pollination by fungus gnat might have evolved repeatedly across the genus (Fig. 4). These results indicate that adaptation to pollination by fungus gnat has driven the repeated evolution of a suite of floral characters in *Euonymus*.

### *Evolution of floral characters in the genus* Euonymus *in relationship to pollination*

The morphological fit between flowers and pollinating animals is essential to achieving effective and selective pollen transportation by pollinators (Mayfield *et al.*, 2001; Castellanos *et al.*, 2004; Pauw, 2006; Shuttleworth *et al.*, 2017). Pollinator shift is therefore expected to be associated with morphological changes in flowers (Muchhala, 2007). Our results support the hypothesis that the short stamens shared by some of the fungus gnat-pollinated plants are an adaptation to pollen transfer by fungus gnats (Mochizuki and Kawakita, 2018). In contrast, the exposed nectaries and flat flowers characteristic of fungus gnat-pollinated plants (Okuyama *et al.*, 2004; Mochizuki and Kawakita, 2018) were not tested, because they are already shared by many plants of the Celastraceae, including the genus *Euonymus*. In *Mitella*, the entire flower shape evolves to be flat when shifts in pollinator occur from non-fungus gnat insects to fungus gnats (Okuyama *et al.*, 2008). The parallel adaptation in flower morphology observed in the two genera *Euonymus* and *Mitella* suggests that consistent selective pressure imposed by fungus gnats exists. However, the short stamen is a character not unique to fungus gnat-pollinated plants in the genus *Euonymus*, because bee-pollinated *E. alatus* and *E. planipes* and beetle-pollinated *E. oxyphyllus* also possess short stamens (Fig. 1). In these species, pollinating insects were relatively small compared with those visiting *E. sieboldianus* and *E. japonicus* with long stamens and were observed to carry pollen mainly on the ventral side of the thorax and abdomen. This suggests that the short stamen is also functional in pollen deposition on small insects other than fungus gnats. Thus, although fungus gnat pollinators select for short stamens, divergence from fungus gnat pollination to other pollination systems does not necessarily accompany a shift in floral morphology.

Pollinator observations and the phylogenetically independent contrast suggested that the red flower colour is tied to pollination by fungus gnats (Tables 1 and 2). To our knowledge, this is the first study to exemplify the evolutionary association of red flower colour and dipteran insects by a rigorous test on phylogeny. The red flower display is generally associated with bird pollination and butterfly pollination systems (Rodríguez-Gironés and Santamaría, 2004; Willmer, 2011), whereas the association with other pollinators has not been explored well (Woodcock *et al.*, 2014; Mochizuki and Kawakita, 2018). One example of the relationship between red flower coloration and entomophilous pollination is the red bowl-shaped floral guild in Mediterranean flora, such as *Anemone* and *Tulipa* (Dafni *et al.*, 1990). The red coloration has been hypothesized to be an adaptation towards *Amphicoma* beetles, because other co-occurring plant species with flowers of a similar shape but different colour are pollinated by bees (Dafni *et al.*, 1990; Martínez-Harms *et al.*, 2012). The above examples of plants pollinated by birds, butterflies and beetles possess relatively bright red flowers that reflect 30–50 % of the light of 600–700 nm with/without UV reflection (Martínez-Harms *et al.*, 2012; Shrestra *et al.*, 2013; Butler and Johnson, 2020). Plants pollinated by saprophagous dipterans via floral mimicry, such as *Amorphophallus*, *Stapelia* and *Jaborosa*, might represent another example of entomophilous reddish flowers, but the flowers are much darker in general (5–15 % reflection; Moré *et al.*, 2013; Chen *et al.*, 2015; du Plessis *et al.*, 2018). The flowers of fungus gnat-pollinated *Euonymus* species reflected 15–40 % of the red light (Fig. 2B); therefore, they are darker than the flowers of plants pollinated by birds and butterflies, but brighter than those pollinated by saprophagous flies. The plants pollinated by other nematoceran flies, such as gall midges, possess flowers of similar colours to those of fungus gnat-pollinated plants; therefore, they might have acquired the red floral display in a similar manner to the fungus gnat-pollinated plants (Luo *et al.*, 2017; Kawakita *et al.*, 2022).

Although the phylogenetically independent contrast analysis indicates that a red floral display is evolutionarily associated with pollination by fungus gnats, this does not necessarily indicate that red flower coloration attracts fungus gnats. So far, sciarid fungus gnats are reported to be attracted to a yellow colour (Cloyd and Dickinson, 2005), whereas such a colour preference has not been tested in other fungus gnats, including Mycetophilidae (see Mochizuki and Kawakita, 2018). Another possible explanation resulting from field observations is that the red coloration functions to filter out undesired visitors, such as bees (Lunau *et al.*, 2011). In the sympatric, co-flowering population of *E. planipes* and *E. tricarpus* on Rishiri Island, the fungus gnat-pollinated *E. tricarpus* was never visited by bees despite many of the visitors, including fungus gnats, being shared between the two species (Supplementary Data Table S3). Visitation by bees was also observed in other white-flowered species but was almost never observed in red-flowered species. As discussed above, the spectral reflection pattern of the studied red flowers is similar to those of bird-pollinated plants that generally reflect light of long wavelengths, without UV reflection (Fig. 2B). Unlike the bee-pollinated red flowers that reflect UV, bird-pollinated red flowers mostly absorb UV light and are thus difficult for bees and many other insects to discriminate from the background foliage (Troje, 1993; Briscoe and Chittka, 2001; Lunau *et al.*, 2011). These observations indicate that the red coloration might play a role in filtering out undesired visitors, such as bees (Lunau *et al.*, 2011). Such an adaptation to avoid visitation by bees, which are major exploiters of pollen and nectar, is often used to interpret the predominance of red flowers in bird-pollinated plants (Raven, 1972; Rodríguez-Gironés and Santamaría, 2004; Lunau *et al.*, 2011). Yet, this hypothesis has never been applied to red-coloured flowers of non-bird-pollinated plants and should be tested in future studies.

The floral scent compositions of white-flowered species are more diverse than those of red-flowered species (Supplementary Data Table S4), but because SPME fibres possess less quantitativity than Tenax, it is likely that the amount of acetoin could be underestimated (Povolo and Contarini, 2003). Another caution is that terpenoids did not appear frequently in samples using Tenax (Supplementary Data Table S4); therefore, in this study, the analysis of floral scent of *E. atropurpureus* using only Tenax might not be sufficient for a full exploration of the floral scent of this species. The abundance of monoterpenes in floral scents of white-flowered species suggests a generalist or food-seeking bee pollination system (Dobson, 2006). In contrast, the similarity in floral scent of red-flowered species suggests a convergence in floral scent towards pollination by fungus gnats (Fig. 3). Acetoin seems to be a key compound, because other compounds were generally present in only trace amounts or were not detected constantly among the samples as indicated by a large s.d. (Table 2). The floral odour of *E. laxiflorus* was distinct from other red-flowered species, in that it did not possess acetoin. In concert with the UV reflection in *E. laxiflorus* flowers, the mechanism of attracting pollinators might be different from that of other red-flowered fungus gnat-pollinated species, but the reason is currently unclear.

Acetoin is a compound associated with the fermentation process and is commonly found in fermented dairy products, such as yogurt (Xiao and Lu, 2014), and in rotting fruits (Stensmyr *et al.*, 2003), or during the fermentation process of bacteria in soils in nature (Turinsky *et al.*, 2000). Cases where acetoin is the dominant compound in floral scent are rare (Knudsen *et al.*, 2006). In systems where plants emit scent mimicking rotting fruit or yeast, acetoin is reported to account for 10–50 % of floral scent (Goodrich *et al.*, 2006; Vlasáková *et al.*, 2008; Goodrich and Raguso, 2009; Martos *et al.*, 2015; Heiduk *et al.*, 2017; Gottsberger *et al.*, 2021). In these systems, floral scent usually contains other compounds indicative of fermentation, such as 2- and 4-carbon aliphatic compounds (Stökl *et al.*, 2010). The floral scent of *E. melananthus* contained butanediol isomers and 3-methyl-1-butanol, with 2-heptanone and several sesquiterpene compounds, which is similar to the scent of *Asimina* species using fermentation mimicry (Goodrich and Raguso, 2009). However, as it is unknown whether fungus gnats are attracted to fermenting materials, it is unclear whether the fungus gnat-pollinated *Euonymus* species use fermentation mimicry.

Pollination by fungus gnats is often believed to involve mushroom mimicry (Vogel and Martens, 2000; Willmer, 2011), and some species of the frequent pollinator genus *Neoempheria* are considered to feed on fungi or decaying plant materials (Jakovlev, 2011; Sueyoshi, 2014). However, the fungus gnat-pollinated *Euonymus* species do not seem to use mushroom mimicry, because we did not find compounds suggestive of mushrooms, such as 1-octen-3-ol (Combet *et al*., 2006). This is also true in the other genera, such as *Mitella*, *Arisaema* and *Corybas*, where floral odour compounds include monoterpenes, such as linalool (Okamoto *et al.*, 2015), the aliphatic aldehydes nonanal and decanal (Vogel and Martens, 2000), and heptanal, β-pinene, 1-octanol and l-α-terpineol (Han *et al.*, 2022), respectively.

Further study is needed to draw a whole picture of adaptation in floral scent in fungus gnat-pollinated plants. Also, an effort is needed to clarify the life histories of the fungus gnats involved in the pollination of these plants, in order to understand how floral volatiles prompt these insects to visit flowers.

### Fungus gnat pollination syndrome

Traditional pollination syndromes represent convergent floral adaptations associated with specific types of pollinators, which in turn provide predictions of pollinators based on floral traits (Fenster *et al.*, 2004; Dellinger, 2020). The pollination syndrome hypothesis has been examined by investigating the association between pollinators and floral characteristics, with a focus on the specific plant lineage (Wilson *et al.*, 2004; Smith *et al.*, 2008; Martén-Rodríguez *et al.*, 2009; Johnson, 2013; Abrahamczyk *et al.*, 2017; Lagomarsino *et al.*, 2017; Smith and Kriebel, 2018), the specific pollinator guild (Pauw, 2006; Quintero *et al.*, 2017; Serrano-Serrano *et al.*, 2017; Garcia *et al.*, 2022; Pauw, 2022) or the pattern within regional communities (Momose *et al.*, 1998; Hingston and McQuillan, 2000; Johnson, 2010; Danieli-Silva *et al*., 2012; Wang *et al.*, 2020) and across communities (Ollerton *et al.*, 2009; Rosas-Guerrero *et al.*, 2014; Ashworth *et al.*, 2015; Dellinger, 2020).

In our previous study, we determined that several unrelated plants sharing dark red, flat-shaped flowers with a short stamen and exposed nectary are pollinated by fungus gnats and hypothesized that the shared floral characteristics are a syndrome associated with pollination by fungus gnats (for detailed information, see Mochizuki and Kawakita, 2018). The observed phylogenetic association between floral characters and pollination by fungus gnats (Table 2) suggests that the red floral colour, short stamen and acetoin belong to a syndrome reflecting evolutionary specialization towards fungus gnat pollinators (Fenster *et al.*, 2004; Rosas-Guerrero *et al.*, 2014). However, the fungus gnat-pollinated plants in the genus *Euonymus* received visits and pollination contributions by other insect taxa (Table 1). Such apparent generalization in pollination is common in plants with traditional syndromes (Ollerton *et al.*, 2009). In concert with the recognition that the floral traits might evolve in response to selective pressure exerted by multiple pollinators and antagonists (Aigner, 2001), the idea of a pollination syndrome is considered to include not only specialization for primary pollinators but adaptive generalization towards other visitors (Dellinger *et al.*, 2019; Gavrutenko *et al.*, 2020; Ohashi *et al.*, 2021). As discussed in the previous section, floral traits of fungus gnat-pollinated *Euonymus* plants might reflect either adaptation to attract the primary pollinator fungus gnats and/or to discourage visitation by non-beneficial insects. To clarify whether the combination of floral traits associated with fungus gnats is an extended syndrome resulting from comprehensive adaptation to both pollinators and antagonists, it will be important to examine the ecological role of each floral trait (Fenster *et al.*, 2004). We believe that entangling the function of each trait in the fungus gnat-pollination systems would provide a better understanding of the recent discussion about the pollination syndrome.

We should note that not all the plants pollinated by fungus gnats share a complete set of these floral traits (Mochizuki and Kawakita, 2018). This is presumably attributable to the diverse way in which plant species use fungus gnats as pollinators. For example, floral architecture should be different between plants using a pseudo-copulatory system and a rewarding system to locate pollen effectively to the pollinator body (e.g. Reiter *et al.*, 2019). The requirement for the adaptation to fungus gnat pollination might also be different between nectar-rewarding *Mitella* and *Euonymus*: petal colour is green or red and acetoin does not appear in *Mitella* (Okuyama *et al.*, 2008; Okamoto *et al.*, 2015), although the precise reason for the difference in floral traits is unclear. We believe that the evolutionary pattern observed in *Euonymus* can be observed in some systems in which plants reward fungus gnats with nectar. For example, *Aucuba japonica* (Garryaceae) and *Micranthes fusca* (Saxifragaceae) share similar floral traits to fungus gnat-pollinated *Euonymus* plants and have congeners that possess whitish flowers with long stamens (K. Mochizuki, pers. obs.).

Finally, despite the realization that dipteran insects are significant pollinators of flowering plants (Ssymank *et al.*, 2008; Orford *et al.*, 2015), the lack of phylogenetic study hinders our understanding of the role of dipteran insects as a driver of floral evolution (but see Okuyama *et al.*, 2008; Garcia *et al.*, 2022). The current categorization of a pollination syndrome in plants pollinated by dipteran insects should be improved (Larson *et al.*, 2001; Labandeira, 2005). Even the classical sapromyiophilous pollination systems are now considered to include several distinct types based on floral scent; they are mimics of dung, urine and dead bodies (van der Niet *et al.*, 2011; Jürgens *et al.*, 2013; Johnson and Schiestl, 2016). Furthermore, given that plants exhibit distinct adaptation towards specific groups of dipteran insects, especially in floral scent such as kleptomyiophily reported in Apocynaceae and Aristolochiaceae (Heiduk *et al.*, 2017), aphid mimicry in several orchid genera (Stökl *et al.*, 2011; Jiang *et al.*, 2020) and fungus gnat pollination systems observed in five unrelated plant families (Mochizuki and Kawakita, 2018; this study), the order Diptera clearly includes several functional groups (Raguso, 2020). The ~70 families of dipteran insects involved in pollination have a diverse larval and adult life history, which might have different visual and olfactory preferences (Larson *et al.*, 2001). Given the large diversity of pollinating Diptera and the fact that floral scent is of particular importance in the attraction of each pollinating Diptera, the diversity of floral scent in Diptera-pollinated plants might still be underestimated (Johnson and Schiestl, 2016). A close look at the groups of dipteran insects and a use of phylogeny in combination with floral scent analysis might help to shed light on the functional diversity of dipteran pollinators and their role in floral evolution.

## SUPPLEMENTARY DATA

Supplementary data are available at *Annals of Botany* online and consist of the following.

Figure S1: the images of the studied plant and their fungus gnat pollinators. Figure S2: result of floral scent analysis based on NMDS using only the data collected with SPME fibres. Figure S3: result of ancestral reconstruction on flower colour based on maximum parsimony and maximum likelihood using Mesquite. Table S1: summary of field observations conducted in this study. Table S2: list of plant materials, with collection locality, voucher information and GenBank accession numbers of the sequences obtained in this study. Table S3: the number of flower visitors observed at each plant species. Table S4: Floral scent profiles of *Euonymus* spp. and *T. regelii*. Values represent relative amounts (%) expressed as percent peak areas in gas chromatograms (mean ± standard deviation) and the hyphen in the column indicates the absence. Most of the samples were collected using SPME, and for those using Tenax are indicated in parentheses after the species name.

mcad081_suppl_Supplementary_Figures

mcad081_suppl_Supplementary_Tables

## Data Availability

The data is presented in the supplementary files and the DRYAD data repository (https://doi.org/10.5061/dryad.msbcc2g46).
