# Supplementary material for: Adaptation to pollination by fungus gnats underlies the evolution of pollination syndrome in the genus Euonymus
Source: Ann Bot. 2023 Aug 23;132(2):319–33. doi: 10.1093/aob/mcad081 (PMC10583214; doi:10.1093/aob/mcad081)
Supplement: mcad081_suppl_Supplementary_Figures [file mcad081_suppl_supplementary_figures.docx]

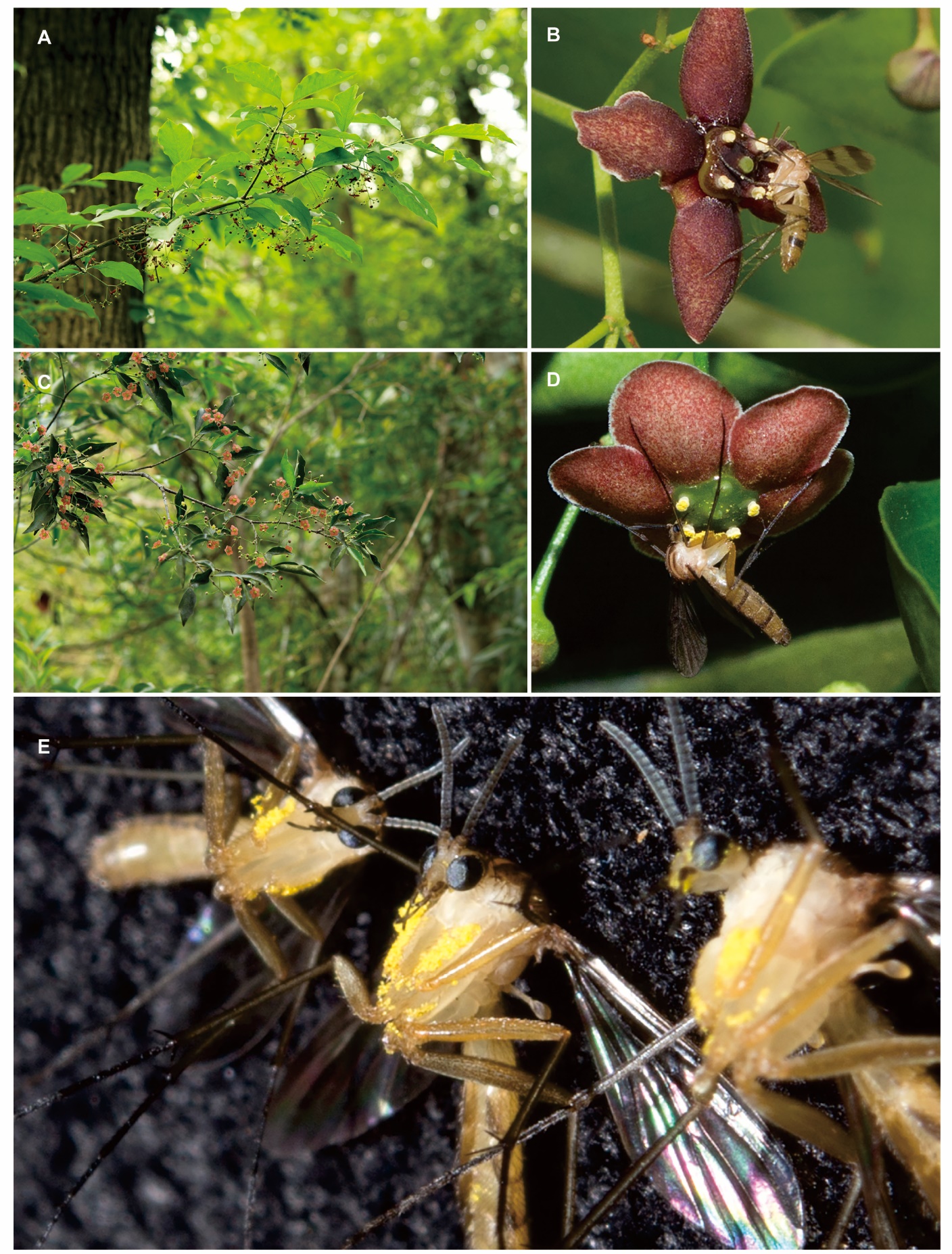
**Supplementary information for [23050] Mochizuki et al., “ Adaptation to pollination by fungus gnats underlies the evolution of pollination syndrome in the genus *Euonymus*”**

Figure S1

The images of the studied plant and their fungus gnat pollinators. (A) The flowering branch of *E. atropurpureus*. (B) *Neoempheria* sp. 5 feeding on the nectar at *E. atropurpureus*. (C) The flowering branch of *E. laxiflorus*. (D) *Neoempheria* sp. 2 feeding on the nectar at *E. laxiflorus*. (E) *Neoempheria* sp. 2 individuals carrying yellow pollen grains of *E. laxiflorus* on their coxa.


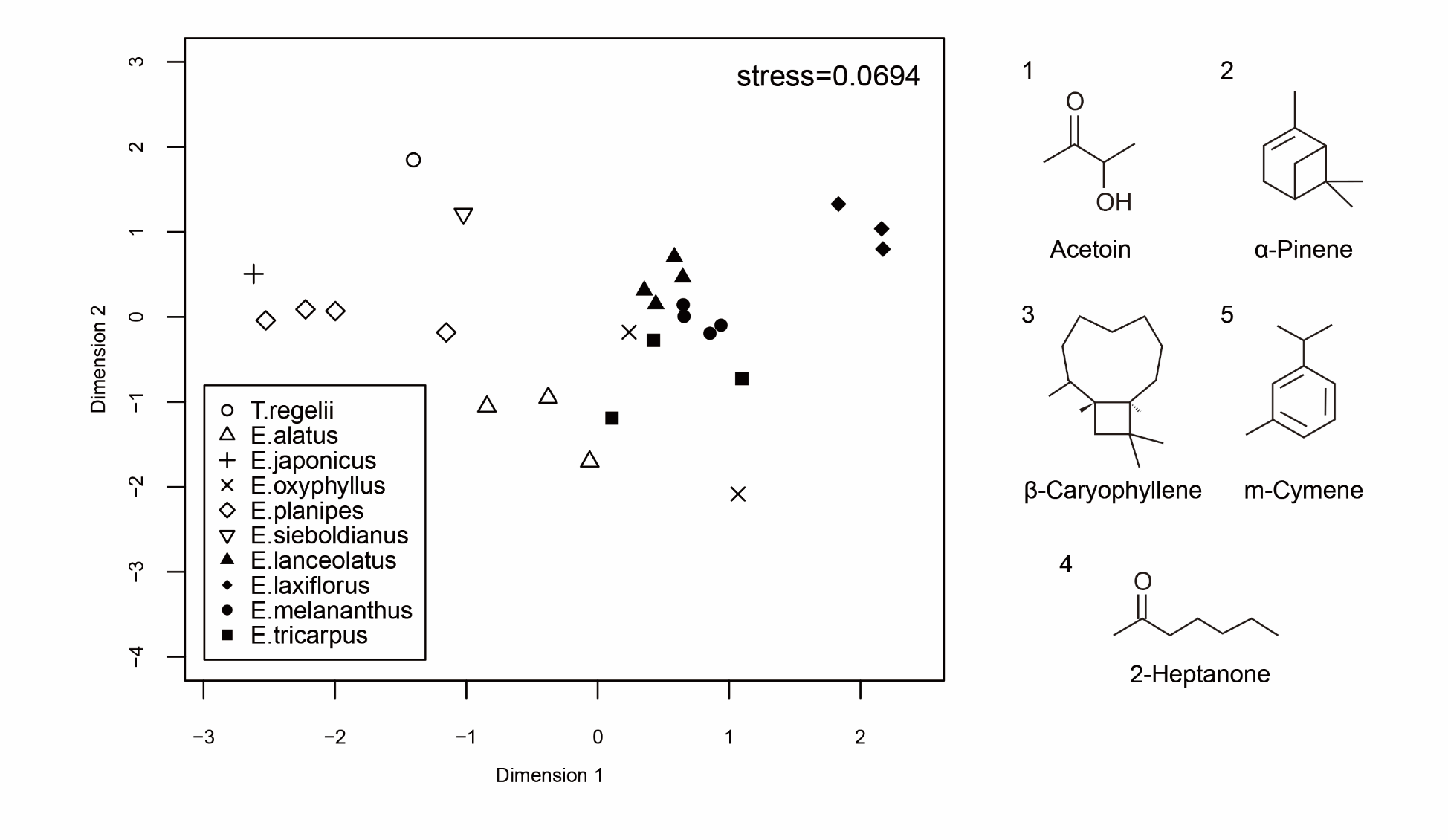
Figure S2

Result of floral scent analysis based on NMDS using only the data collected with SPME fibres. The compositions of the floral scent were significantly different between red- and white- flowered species (PERMANOVA, F=5.25, R^2^=0.18, p=0.001). Acetoin was the primary explanatory compound (SIMPER contribution 14.8%) of the difference of floral scent between white- and red- flowered species, followed by α-pinene, β-caryophyllene, 2-heptanone and m-cymene, which composed 39.5% of the total explanatory variables.


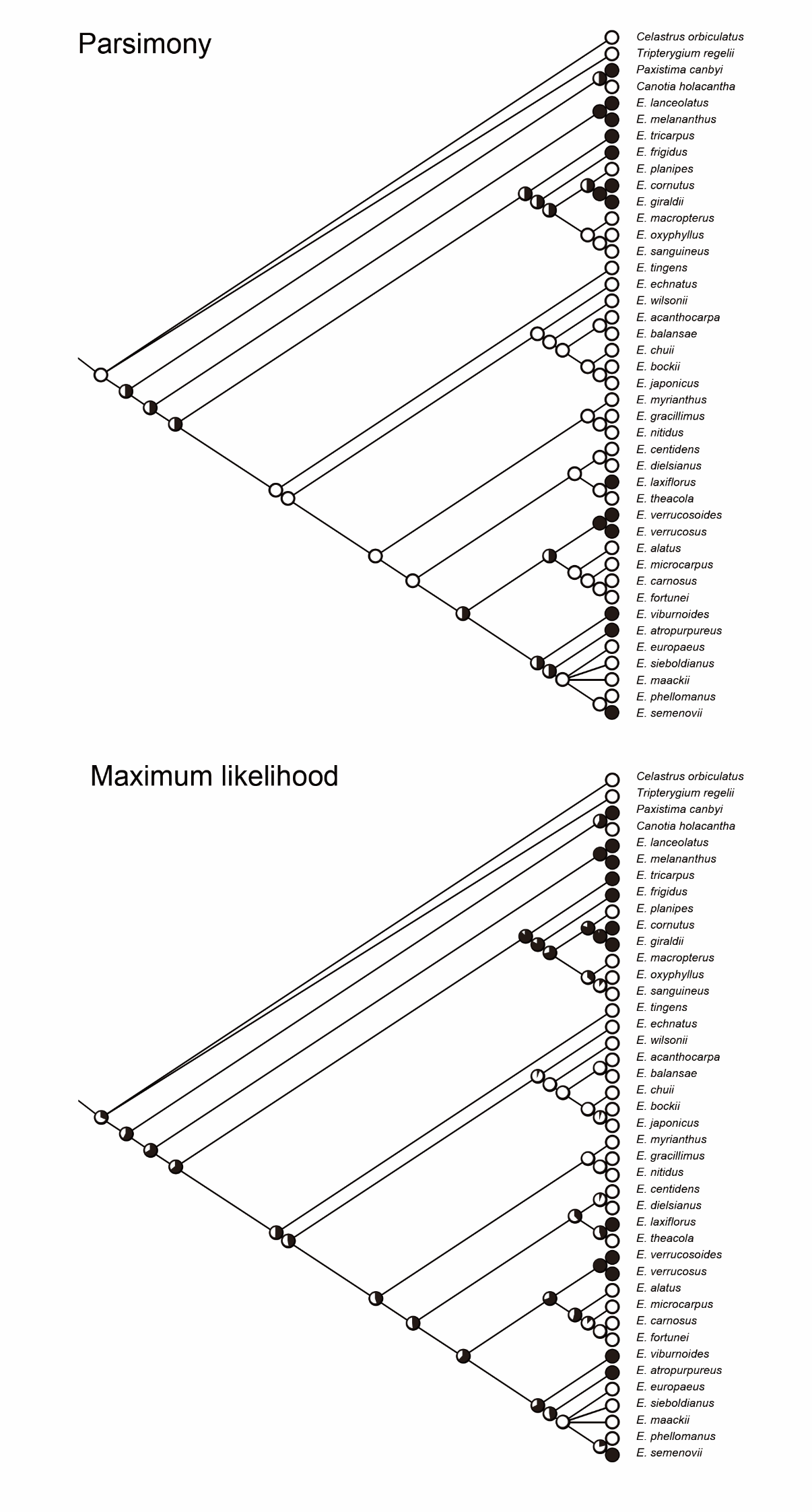


Figure S3

Result of ancestral reconstruction on flower colour based on maximum parsimony and maximum likelihood using MESQUITE.
