# Supplementary material for: Adaptation to pollination by fungus gnats underlies the evolution of pollination syndrome in the genus Euonymus
Source: Ann Bot. 2023 Aug 23;132(2):319–33. doi: 10.1093/aob/mcad081 (PMC10583214; doi:10.1093/aob/mcad081)
Supplement: mcad081_suppl_Supplementary_Tables [file mcad081_suppl_supplementary_tables.docx]

**Supplementary tables for [23050] Mochizuki et al., “ Adaptation to pollination by fungus gnats underlies the evolution of pollination syndrome in the genus *Euonymus*”**

Table S1. Summary of field observations conducted in this study.

| Plant species | Date | Duration | Location |
| --- | --- | --- | --- |
| *Euonymus alatus* | 20 May 2016 | 1000–1930 | Mt. Ryozensan, Shiga pref., Japan |
|  | 22 May 2016 | 1100–2200 | Kibune, Kyoto pref., Japan |
| *E. atropurpureus* | 21 Jun. 2019 | 1400–1900 | Somme Woods, Northbrook, Illinois, USA |
|  | 23 Jun. 2019 | 1000–1900 | Somme Woods, Northbrook, Illinois, USA |
|  | 22 Jun. 2019 | 1430–1930 | LaBagh Woods, North Park, Illinois, USA |
| *E. sieboldianus* | 10 Jul. 2017 | 1000–2000 | Mt. Hakusan, Ishikawa pref., Japan |
|  | 20 May 2018 | 1500–1600 | Fujikawaguchiko-cho, Yamanashi pref., Japan |
|  | 22 May 2018 | 0900–1000 | Koishikawa Botanical Garden, Tokyo, Japan |
|  | 24 Jul. 2019 | 1300–1700 | Mt. Hakusan, Ishikawa pref., Japan |
| *E. japonicus* | 10 Jun. 2015 | 1300–1600 | Tomogashima island, Wakayama pref., Japan |
|  | 26 Jun. 2016 | 0930–1200 | Shimane-cho, Shimane pref., Japan |
|  | 26 Jun. 2016 | 1900–2200 | Daisen-cho, Tottori pref., Japan |
| *E. laxiflorus* | 23, 24, 25 May 2019 | 0900–2100 | Cuku-li, Pinglin District, Taiwan |
| *E. oxyphyllus* | 13 May 2015 | 1200–1300 | Mt. Yokoyamadake, Fukui pref., Japan |
|  | 19 May 2017 | 0945–1920 | Mt. Yashagaike, Fukui pref., Japan |
|  | 15 Jun. 2017 | 1000–1940 | Shiramine, Ishikawa pref., Japan |
| *E. planipes* | 10 Jun. 2016 | 0930–2100 | Oniwaki, Rishiri island, Hokkaido, Japan |
|  | 11 Jun. 2016 | 1330–1715 | Oniwaki, Rishiri island, Hokkaido, Japan |
|  | 12 Jun. 2016 | 0900–2000 | Oniwaki, Rishiri island, Hokkaido, Japan |
| *Tripterigium wilfordii* | 7 Jul. 2016 | 0900–0945, 1700–2100 | Mt. Hakusan, Ishikawa pref., Japan |
|  | 8 Jul. 2016 | 1000–1130 | Mt. Hakusan, Ishikawa pref., Japan |
|  | 13 Jul. 2016 | 0900–1200 | Mt. Hakusan, Ishikawa pref., Japan |
|  | 24 Jul. 2019 | 0930–1000 | Mt. Hakusan, Ishikawa pref., Japan |
|  | 24 Jul. 2019 | 1500–1630 | Shiramine, Ishikawa pref., Japan |

Table S2. List of plant materials with collection locality, voucher information and GenBank accession numbers of the sequences obtained in this study.

| Plant species | Locality | Voucher ID | GenBank accession number | |
| --- | --- | --- | --- | --- |
|  |  |  | ITS | ETS |
| *Euonymus atropurpureus* | Somme Woods, Northbrook, Illinois, USA | TI00108355 | LC662753 | LC662748 |
| *E. japonicus* | Shimane-cho, Shimane pref., Japan | TI00108356 | HQ393700 | LC663319 |
| *E. laceolatus* | Shiramine, Ishikawa pref., Japan | TI00108357 | LC662749 | LC662744 |
| *E. melananthus* | Shiramine, Ishikawa pref. | TI00108358 | LC662750 | LC662745 |
| *E. planipes* | Oniwaki, Rishiri island, Hokkaido, Japan | TI00108359 | LC662751 | LC662746 |
| *E. tricarpus* | Oniwaki, Rishiri island, Hokkaido, Japan | TI00108360 | LC662752 | LC662747 |

Table S3. The number of flower visitors observed at each plant species.

| Plant species | Functional group | Family | Species | Frequency |
| --- | --- | --- | --- | --- |
| *Euonymus atropurpureus* | Fungus gnat | Mycetophilidae | *Neoempheria* sp. 5 | 8 |
|  | Fungus gnat | Mycetophilidae | *Neoempheria* sp. 6 | 2 |
|  | Fungus gnat | Mycetophilidae | Unknown, not captured | 4 |
|  |  |  |  |  |
| *E. lanceolatus* | Fungus gnat | Mycetophilidae | *Epicypta* sp. 1 | 1 |
|  | Fungus gnat | Mycetophilidae | *Epicypta* sp. 2 | 2 |
|  | Fungus gnat | Mycetophilidae | *Epicypta* sp. 3 | 1 |
|  | Fungus gnat | Mycetophilidae | *Mycetophila* sp. 1 | 1 |
|  | Fungus gnat | Mycetophilidae | *Neoempheria* sp. 1 | 13 |
|  | Fungus gnat | Mycetophilidae | Unknown, not captured | 58 |
|  | Fungus gnat | Sciaridae | spp. | 4 |
|  | Other Nematocera | Cecidomyiidae | sp. 1 | 1 |
|  | Other Nematocera | Cecidomyiidae | Unknown, not captured | 3 |
|  | Other Nematocera | Chironomidae | sp. 1 | 1 |
|  | Non-syrphid Brachycera | Drosophilidae | sp. 6 | 16 |
|  | Non-syrphid Brachycera | Drosophilidae | sp. 7 | 2 |
|  | Non-syrphid Brachycera | Drosophilidae | Unknown, not captured | 11 |
|  | Ant | Formicidae | sp. 4 | 8 |
|  | Ant | Formicidae | Unknown, not captured | 10 |
|  | Other short-proboscised arthropods | Entomobryidae | sp. | 2 |
|  | Other short-proboscised arthropods | Rhaphidophoridae | sp. | 1 |
|  | Other long-proboscised arthropods | Miridae | *Apolygus* sp. | 1 |
|  |  |  |  |  |
| *E. laxiflorus* | Fungus gnat | Keroplatidae | *Cerotelion* sp. 1 | 1 |
|  | Fungus gnat | Keroplatidae | *Cerotelion* sp. 2 | 4 |
|  | Fungus gnat | Keroplatidae | *Euceroplatus* sp. | 4 |
|  | Fungus gnat | Keroplatidae | *Proceroplatus* sp. | 36 |
|  | Fungus gnat | Mycetophilidae | *Epicypta* sp. 6 | 1 |
|  | Fungus gnat | Mycetophilidae | Genus unknown sp. 4 | 1 |
|  | Fungus gnat | Mycetophilidae | Genus unknown sp. 7 | 1 |
|  | Fungus gnat | Mycetophilidae | *Neoempheria* sp. 2 | 10 |
|  | Fungus gnat | Mycetophilidae | *Neoempheria* sp. 3 | 8 |
|  | Fungus gnat | Mycetophilidae | *Neoempheria* sp. 4 | 6 |
|  | Fungus gnat | Mycetophilidae | *Neuratelia* sp. | 2 |
|  | Fungus gnat | Sciaridae | spp. | 41 |
|  | Cranefly | Tipulidae | sp. 3 | 1 |
|  | Other Nematocera | Cecidomyiidae | sp. 3 | 1 |
|  | Other Nematocera | Cecidomyiidae | sp. 6 | 1 |
|  | Other Nematocera | Ceratopogonidae | sp. 1 | 24 |
|  | Other Nematocera | Ceratopogonidae | sp. 2 | 4 |
|  | Other Nematocera | Chironomidae | sp. 2 | 3 |
|  | Other Nematocera | Chironomidae | sp. 4 | 3 |
|  | Non-syrphid Brachycera | Dolichopodidae | sp. 1 | 1 |
|  | Non-syrphid Brachycera | Drosophilidae | sp. 2 | 1 |
|  | Non-syrphid Brachycera | Drosophilidae | sp. 3 | 2 |
|  | Non-syrphid Brachycera | Drosophilidae | sp. 4 | 1 |
|  | Non-syrphid Brachycera | Drosophilidae | sp. 5 | 1 |
|  | Non-syrphid Brachycera | Empididae | sp. 3 | 1 |
|  | Non-syrphid Brachycera | Empididae | sp. 4 | 1 |
|  | Non-syrphid Brachycera | Ephydridae | sp. 2 | 7 |
|  | Non-syrphid Brachycera | Heleomyzidae | sp. 1 | 4 |
|  | Non-syrphid Brachycera | Lauxaniidae | sp. 2 | 9 |
|  | Non-syrphid Brachycera | Lauxaniidae | sp. 3 | 3 |
|  | Non-syrphid Brachycera | Muscidae | sp. 6 | 1 |
|  | Non-syrphid Brachycera | Muscidae | sp. 7 | 1 |
|  | Non-syrphid Brachycera | Muscidae | sp. 9 | 4 |
|  | Non-syrphid Brachycera | Phoridae | sp. 4 | 1 |
|  | Non-syrphid Brachycera | Platystomatidae | sp. | 3 |
|  | Non-syrphid Brachycera | Tachinidae | sp. 1 | 3 |
|  | Non-syrphid Brachycera | Tachinidae | sp. 3 | 1 |
|  | Non-syrphid Brachycera | Tachinidae | sp. 4 | 1 |
|  | Non-syrphid Brachycera | Tephritidae | sp. | 1 |
|  | Non-syrphid Brachycera | Unknown | not captured | 1 |
|  | Bee | Halictidae | sp. 7 | 1 |
|  | Other Hymenoptera | Braconidae | sp. 2 | 1 |
|  | Other Hymenoptera | Ichneumonidae | sp. 5 | 2 |
|  | Other Hymenoptera | Platygastridae | sp. | 1 |
|  | Other Hymenoptera | Unknown | sp. 1 | 1 |
|  | Other Hymenoptera | Unknown | sp. 2 | 1 |
|  | Lepidoptera | Tortricidae | sp. | 1 |
|  |  |  |  |  |
| *E. melananthus* | Fungus gnat | Mycetophilidae | *Anatella* sp. 2 | 2 |
|  | Fungus gnat | Mycetophilidae | Genus unknown sp. 1 | 1 |
|  | Fungus gnat | Mycetophilidae | Genus unknown sp. 5 | 1 |
|  | Fungus gnat | Mycetophilidae | Genus unknown sp. 6 | 1 |
|  | Fungus gnat | Mycetophilidae | *Mycomya* sp. 1 | 1 |
|  | Fungus gnat | Mycetophilidae | *Mycomya* sp. 2 | 2 |
|  | Fungus gnat | Mycetophilidae | *Mycomya* sp. 3 | 1 |
|  | Fungus gnat | Mycetophilidae | *Mycomya* sp. 4 | 1 |
|  | Fungus gnat | Mycetophilidae | *Neoempheria* sp. 1 | 2 |
|  | Fungus gnat | Mycetophilidae | Unknown, not captured | 4 |
|  | Fungus gnat | Sciaridae | spp. | 5 |
|  | Other Nematocera | Cecidomyiidae | sp. 5 | 4 |
|  | Non-syrphid Brachycera | Drosophilidae | sp. 6 | 2 |
|  | Non-syrphid Brachycera | Drosophilidae | sp. 7 | 1 |
|  | Non-syrphid Brachycera | Muscidae | sp. 12 | 1 |
|  | Non-syrphid Brachycera | Unknown | sp. 2 | 1 |
|  | Coleoptera | Cantharidae | sp. 1 | 1 |
|  | Coleoptera | Cerambycidae | *Pidonia* sp. 2 | 1 |
|  | Coleoptera | Elateridae | sp. 3 | 1 |
|  | Ant | Formicidae | sp. 4 | 1 |
|  | Lepidoptera | Gracillariidae | sp. | 1 |
|  | Lepidoptera | Pyralidae | sp. 1 | 1 |
|  | Other short-proboscised arthropods | Hemerobiidae | sp. | 2 |
|  |  |  |  |  |
| *E. tricarpus* | Fungus gnat | Mycetophilidae | *Boletina* sp. 1 | 14 |
|  | Fungus gnat | Mycetophilidae | *Boletina* sp. 2 | 9 |
|  | Fungus gnat | Mycetophilidae | *Brevicornu* sp. 1 | 2 |
|  | Fungus gnat | Mycetophilidae | *Brevicornu* sp. 2 | 1 |
|  | Fungus gnat | Mycetophilidae | *Epicypta* sp. 4 | 1 |
|  | Fungus gnat | Mycetophilidae | *Epicypta* sp. 5 | 2 |
|  | Fungus gnat | Mycetophilidae | *Mycetophila* sp. 2 | 1 |
|  | Fungus gnat | Mycetophilidae | Genus unknown sp. 3 | 1 |
|  | Fungus gnat | Sciaridae | spp. | 662 |
|  | Cranefly | Limoniidae | sp. 1 | 4 |
|  | Cranefly | Limoniidae | sp. 2 | 4 |
|  | Other Nematocera | Bibionidae | sp. 1 | 1 |
|  | Other Nematocera | Bibionidae | sp. 2 | 4 |
|  | Other Nematocera | Cecidomyiidae | sp. 4 | 1 |
|  | Other Nematocera | Ceratopogonidae | sp. 4 | 12 |
|  | Other Nematocera | Ceratopogonidae | sp. 5 | 2 |
|  | Other Nematocera | Chironomidae | sp. 5 | 2 |
|  | Other Nematocera | Psychodidae | sp. | 2 |
|  | Non-syrphid Brachycera | Acroceridae | sp. | 1 |
|  | Non-syrphid Brachycera | Agromyzidae | sp. 2 | 2 |
|  | Non-syrphid Brachycera | Anthomyiidae | sp. 5 | 8 |
|  | Non-syrphid Brachycera | Drosophilidae | sp. 8 | 1 |
|  | Non-syrphid Brachycera | Empididae | sp. 1 | 1 |
|  | Non-syrphid Brachycera | Empididae | sp. 10 | 1 |
|  | Non-syrphid Brachycera | Empididae | sp. 12 | 1 |
|  | Non-syrphid Brachycera | Empididae | sp. 9 | 1 |
|  | Non-syrphid Brachycera | Muscidae | sp. 11 | 1 |
|  | Non-syrphid Brachycera | Muscidae | sp. 2 | 1 |
|  | Non-syrphid Brachycera | Phoridae | sp. 1 | 1 |
|  | Coleoptera | Cerambycidae | *Chlorophorus japonicus* | 1 |
|  | Coleoptera | Elateridae | *Ectinus dahuricus* | 1 |
|  | Coleoptera | Elateridae | sp. 1 | 5 |
|  | Coleoptera | Elateridae | sp. 2 | 1 |
|  | Coleoptera | Lycidae | sp. 1 | 1 |
|  | Coleoptera | Oedemeridae | sp. 1 | 2 |
|  | Coleoptera | Oedemeridae | sp. 2 | 1 |
|  | Ant | Formicidae | sp. 6 | 7 |
|  | Other Hymenoptera | Braconidae | sp. 3 | 1 |
|  | Other Hymenoptera | Braconidae | sp. 4 | 1 |
|  | Other Hymenoptera | Braconidae | sp. 5 | 1 |
|  | Other Hymenoptera | Eurytomidae | sp. | 1 |
|  | Other Hymenoptera | Ichneumonidae | sp. 10 | 1 |
|  | Other Hymenoptera | Ichneumonidae | sp. 8 | 1 |
|  | Other Hymenoptera | Proctotrupidae | sp. | 1 |
|  | Other Hymenoptera | Tenthredinidae | sp. 1 | 1 |
|  | Lepidoptera | Unknown | sp. | 2 |
|  |  |  |  |  |
| *E. alatus* | Fungus gnat | Sciaridae | spp. | 2 |
|  | Other Nematocera | Ceratopogonidae | sp. 3 | 1 |
|  | Cranefly | Tipulidae | sp. 2 | 1 |
|  | Hoverfly | Syrphidae | sp. 10 | 2 |
|  | Hoverfly | Syrphidae | sp. 11 | 1 |
|  | Hoverfly | Syrphidae | sp. 12 | 1 |
|  | Hoverfly | Syrphidae | sp. 14 | 1 |
|  | Hoverfly | Syrphidae | sp. 15 | 1 |
|  | Non-syrphid Brachycera | Agromyzidae | sp. 1 | 1 |
|  | Non-syrphid Brachycera | Calliphoridae | sp. 5 | 1 |
|  | Non-syrphid Brachycera | Chloropidae | sp. | 1 |
|  | Non-syrphid Brachycera | Dolichopodidae | sp. 2 | 1 |
|  | Non-syrphid Brachycera | Empididae | sp. 5 | 1 |
|  | Non-syrphid Brachycera | Empididae | sp. 6 | 1 |
|  | Non-syrphid Brachycera | Empididae | sp. 7 | 1 |
|  | Non-syrphid Brachycera | Lauxaniidae | sp. 4 | 7 |
|  | Non-syrphid Brachycera | Lauxaniidae | sp. 5 | 1 |
|  | Non-syrphid Brachycera | Lauxaniidae | sp. 6 | 5 |
|  | Non-syrphid Brachycera | Lauxaniidae | sp. 7 | 2 |
|  | Non-syrphid Brachycera | Muscidae | sp. 8 | 1 |
|  | Non-syrphid Brachycera | Stratiomyidae | sp. 1 | 1 |
|  | Non-syrphid Brachycera | Stratiomyidae | sp. 2 | 3 |
|  | Non-syrphid Brachycera | Tachinidae | sp. 1 | 1 |
|  | Non-syrphid Brachycera | Tachinidae | sp. 5 | 1 |
|  | Non-syrphid Brachycera | Unknown | sp. 3 | 1 |
|  | Coleoptera | Anthicidae | sp. | 1 |
|  | Coleoptera | Cantharidae | *Podabrus heydeni* | 1 |
|  | Coleoptera | Cantharidae | sp. 1 | 6 |
|  | Coleoptera | Cantharidae | sp. 2 | 3 |
|  | Coleoptera | Cerambycidae | *Dinoptera minuta* | 1 |
|  | Coleoptera | Cerambycidae | *Pidonia puziloi* | 3 |
|  | Coleoptera | Curculionidae | sp. | 1 |
|  | Coleoptera | Nitidulidae | sp. | 1 |
|  | Ant | Formicidae | sp. 2 | 1 |
|  | Ant | Formicidae | sp. 3 | 5 |
|  | Bee | Andrenidae | sp. 2 | 2 |
|  | Bee | Apidae | *Ceratina* sp.1 | 2 |
|  | Bee | Apidae | *Ceratina* sp.2 | 2 |
|  | Bee | Apidae | *Nomada* sp. | 1 |
|  | Bee | Halictidae | sp. 1 | 2 |
|  | Bee | Halictidae | sp. 4 | 1 |
|  | Bee | Halictidae | sp. 6 | 1 |
|  | Other Hymenoptera | Braconidae | sp. 1 | 1 |
|  | Other Hymenoptera | Chalcididae | sp. | 1 |
|  | Other Hymenoptera | Ichneumonidae | sp. 11 | 1 |
|  | Other Hymenoptera | Ichneumonidae | sp. 3 | 1 |
|  | Other Hymenoptera | Ichneumonidae | sp. 4 | 2 |
|  | Other Hymenoptera | Ichneumonidae | sp. 6 | 1 |
|  | Other Hymenoptera | Ichneumonidae | sp. 7 | 1 |
|  | Other Hymenoptera | Ichneumonidae | sp. 9 | 1 |
|  | Other Hymenoptera | Tenthredinidae | sp. 1 | 1 |
|  | Other Hymenoptera | Tenthredinidae | sp. 2 | 1 |
|  | Other Hymenoptera | Tenthredinidae | sp. 3 | 1 |
|  | Other Hymenoptera | Tenthredinidae | sp. 4 | 1 |
|  | Other Hymenoptera | Tenthredinidae | sp. 5 | 1 |
|  | Other long-proboscised arthropods | Panorpidae | sp. 1 | 1 |
|  | Other short-proboscised arthropods | Membracidae | sp. | 1 |
|  |  |  |  |  |
| *E. japonicus* | Fungus gnat | Sciaridae | spp. | 6 |
|  | Hoverfly | Syrphidae | sp. 13 | 7 |
|  | Hoverfly | Syrphidae | sp. 6 | 1 |
|  | Non-syrphid Brachycera | Anthomyiidae | sp. 1 | 31 |
|  | Non-syrphid Brachycera | Anthomyiidae | sp. 2 | 1 |
|  | Non-syrphid Brachycera | Anthomyiidae | sp. 3 | 2 |
|  | Non-syrphid Brachycera | Anthomyiidae | sp. 4 | 1 |
|  | Non-syrphid Brachycera | Anthomyiidae | sp. 6 | 5 |
|  | Non-syrphid Brachycera | Calliphoridae | sp. 1 | 1 |
|  | Non-syrphid Brachycera | Calliphoridae | sp. 2 | 2 |
|  | Non-syrphid Brachycera | Calliphoridae | sp. 3 | 4 |
|  | Non-syrphid Brachycera | Calliphoridae | sp. 4 | 1 |
|  | Non-syrphid Brachycera | Calliphoridae | sp. 7 | 1 |
|  | Non-syrphid Brachycera | Coelopidae | *Coelopa frigida* | 3 |
|  | Non-syrphid Brachycera | Ephydridae | sp. 1 | 13 |
|  | Non-syrphid Brachycera | Muscidae | sp. 1 | 1 |
|  | Non-syrphid Brachycera | Muscidae | sp. 10 | 1 |
|  | Non-syrphid Brachycera | Rhiniidae | *Stomorhina obsoleta* | 1 |
|  | Non-syrphid Brachycera | Sarcophagidae | sp. 1 | 1 |
|  | Non-syrphid Brachycera | Sarcophagidae | sp. 2 | 1 |
|  | Non-syrphid Brachycera | Stratiomyidae | sp. 2 | 1 |
|  | Non-syrphid Brachycera | Tachinidae | sp. 6 | 1 |
|  | Coleoptera | Cerambycidae | *Pidonia* sp. 1 | 1 |
|  | Coleoptera | Curculionidae | *Lixus* sp. | 1 |
|  | Coleoptera | Curculionidae | *Phyllobius* sp. | 3 |
|  | Coleoptera | Lycidae | sp. 3 | 1 |
|  | Coleoptera | Scarabaeidae | *Anomala rufocuprea* | 1 |
|  | Coleoptera | Staphylinidae | sp. | 1 |
|  | Coleoptera | Unknown | sp. 1 | 4 |
|  | Ant | Formicidae | sp. 1 | 3 |
|  | Ant | Formicidae | sp. 2 | 5 |
|  | Ant | Formicidae | sp. 3 | 1 |
|  | Ant | Formicidae | sp. 5 | 1 |
|  | Bee | Andrenidae | sp. 3 | 1 |
|  | Bee | Apidae | *Bombus ardens* | 1 |
|  | Bee | Apidae | *Ceratina* sp.2 | 2 |
|  | Bee | Apidae | *Xylocopa appendiculata* | 1 |
|  | Other Hymenoptera | Vespidae | *Anterhynchium* sp. | 8 |
|  | Lepidoptera | Nymphalidae | *Libythea celtis* | 1 |
|  | Lepidoptera | Sessiidae | sp. | 1 |
|  |  |  |  |  |
| *E. oxyphyllus* | Fungus gnat | Mycetophilidae | Genus unknown sp. 2 | 2 |
|  | Fungus gnat | Mycetophilidae | Genus unknown sp. 8 | 1 |
|  | Fungus gnat | Mycetophilidae | *Mycomya* sp. 5 | 1 |
|  | Fungus gnat | Sciaridae | spp. | 8 |
|  | Cranefly | Tipulidae | sp. 1 | 3 |
|  | Other Nematocera | Cecidomyiidae | sp. 2 | 2 |
|  | Other Nematocera | Chironomidae | sp. 3 | 1 |
|  | Other Nematocera | Simuliidae | sp. | 1 |
|  | Non-syrphid Brachycera | Empididae | sp. 8 | 1 |
|  | Non-syrphid Brachycera | Heleomyzidae | sp. 2 | 1 |
|  | Non-syrphid Brachycera | Lauxaniidae | sp. 6 | 1 |
|  | Non-syrphid Brachycera | Lauxaniidae | sp. 7 | 3 |
|  | Non-syrphid Brachycera | Muscidae | sp. 11 | 1 |
|  | Non-syrphid Brachycera | Muscidae | sp. 3 | 2 |
|  | Non-syrphid Brachycera | Muscidae | sp. 4 | 1 |
|  | Non-syrphid Brachycera | Muscidae | sp. 8 | 1 |
|  | Non-syrphid Brachycera | Phoridae | sp. 2 | 1 |
|  | Coleoptera | Cerambycidae | *Pidonia aegrota* | 2 |
|  | Coleoptera | Cerambycidae | *Pidonia puziloi* | 24 |
|  | Other Hymenoptera | Tenthredinidae | sp. 6 | 1 |
|  | Lepidoptera | Adelidae | *Nemophora* sp. | 1 |
|  | Lepidoptera | Pyralidae | sp. 2 | 1 |
|  |  |  |  |  |
| *E. planipes* | Fungus gnat | Keroplatidae | *Orfelia* sp. | 3 |
|  | Fungus gnat | Mycetophilidae | *Anatella* sp. 1 | 2 |
|  | Fungus gnat | Mycetophilidae | *Boletina* sp. 1 | 3 |
|  | Fungus gnat | Mycetophilidae | *Boletina* sp. 2 | 3 |
|  | Fungus gnat | Mycetophilidae | *Boletina* sp. 3 | 1 |
|  | Fungus gnat | Mycetophilidae | *Cordyla* sp. | 1 |
|  | Fungus gnat | Mycetophilidae | *Gnoriste* sp. | 1 |
|  | Fungus gnat | Mycetophilidae | Genus unknown sp. 3 | 2 |
|  | Fungus gnat | Sciaridae | spp. | 189 |
|  | Cranefly | Limoniidae | sp. 1 | 1 |
|  | Cranefly | Limoniidae | sp. 3 | 3 |
|  | Other Nematocera | Bibionidae | sp. 2 | 9 |
|  | Other Nematocera | Cecidomyiidae | sp. 4 | 5 |
|  | Other Nematocera | Ceratopogonidae | sp. 4 | 10 |
|  | Other Nematocera | Psychodidae | sp. | 1 |
|  | Hoverfly | Syrphidae | sp. 11 | 1 |
|  | Hoverfly | Syrphidae | sp. 12 | 1 |
|  | Hoverfly | Syrphidae | sp. 16 | 1 |
|  | Non-syrphid Brachycera | Anthomyiidae | sp. 6 | 1 |
|  | Non-syrphid Brachycera | Empididae | sp. 10 | 2 |
|  | Non-syrphid Brachycera | Empididae | sp. 11 | 1 |
|  | Non-syrphid Brachycera | Empididae | sp. 8 | 2 |
|  | Non-syrphid Brachycera | Empididae | sp. 9 | 4 |
|  | Non-syrphid Brachycera | Muscidae | sp. 11 | 15 |
|  | Non-syrphid Brachycera | Muscidae | sp. 13 | 1 |
|  | Non-syrphid Brachycera | Tachinidae | sp. 7 | 1 |
|  | Coleoptera | Chrysomelidae | sp. | 1 |
|  | Coleoptera | Elateridae | *Anostirus daimio* | 1 |
|  | Coleoptera | Elateridae | sp. 1 | 1 |
|  | Coleoptera | Elateridae | sp. 2 | 3 |
|  | Bee | Andrenidae | sp. 1 | 2 |
|  | Bee | Andrenidae | sp. 2 | 1 |
|  | Bee | Halictidae | sp. 1 | 1 |
|  | Bee | Halictidae | sp. 2 | 6 |
|  | Bee | Halictidae | sp. 3 | 1 |
|  | Bee | Halictidae | sp. 5 | 4 |
|  | Other Hymenoptera | Argidae | sp. | 1 |
|  | Other Hymenoptera | Ichneumonidae | sp. 1 | 1 |
|  | Other Hymenoptera | Ichneumonidae | sp. 12 | 1 |
|  | Other Hymenoptera | Ichneumonidae | sp. 2 | 1 |
|  | Other Hymenoptera | Pompilidae | sp. | 2 |
|  |  |  |  |  |
| *E. sieboldianus* | Hoverfly | Syrphidae | sp. 1 | 1 |
|  | Hoverfly | Syrphidae | sp. 12 | 3 |
|  | Hoverfly | Syrphidae | sp. 16 | 1 |
|  | Hoverfly | Syrphidae | sp. 7 | 1 |
|  | Hoverfly | Syrphidae | sp. 8 | 1 |
|  | Hoverfly | Syrphidae | sp. 9 | 2 |
|  | Non-syrphid Brachycera | Anthomyiidae | sp. 7 | 1 |
|  | Non-syrphid Brachycera | Asilidae | sp. | 1 |
|  | Non-syrphid Brachycera | Calliphoridae | sp. 6 | 4 |
|  | Non-syrphid Brachycera | Drosophilidae | sp. 1 | 1 |
|  | Non-syrphid Brachycera | Empididae | sp. 2 | 1 |
|  | Non-syrphid Brachycera | Lauxaniidae | sp. 1 | 1 |
|  | Non-syrphid Brachycera | Lauxaniidae | sp. 7 | 2 |
|  | Non-syrphid Brachycera | Muscidae | sp. 12 | 44 |
|  | Non-syrphid Brachycera | Muscidae | sp. 5 | 1 |
|  | Non-syrphid Brachycera | Phoridae | sp. 3 | 1 |
|  | Non-syrphid Brachycera | Sarcophagidae | sp. 3 | 1 |
|  | Non-syrphid Brachycera | Sarcophagidae | sp. 4 | 1 |
|  | Non-syrphid Brachycera | Tachinidae | sp. 2 | 2 |
|  | Non-syrphid Brachycera | Tachinidae | sp. 8 | 1 |
|  | Coleoptera | Cantharidae | sp. 1 | 2 |
|  | Coleoptera | Cantharidae | sp. 2 | 1 |
|  | Coleoptera | Carabidae | sp. | 1 |
|  | Coleoptera | Cerambycidae | sp. | 1 |
|  | Coleoptera | Lycidae | sp. 2 | 1 |
|  | Coleoptera | Scarabaeidae | *Rhomborrhina japonica* | 1 |
|  | Coleoptera | Unknown | sp. 2 | 1 |
|  | Ant | Formicidae | sp. 7 | 1 |
|  | Bee | Apidae | *Ceratina* sp.3 | 1 |
|  | Other Hymenoptera | Ichneumonidae | sp. 13 | 1 |
|  | Lepidoptera | Glyphipterigidae | sp. | 2 |
|  | Other long-proboscised arthropods | Panorpidae | sp. 2 | 1 |
|  |  |  |  |  |
| *Tripterygium regelii* | Hoverfly | Syrphidae | sp. 16 | 1 |
|  | Hoverfly | Syrphidae | sp. 2 | 3 |
|  | Hoverfly | Syrphidae | sp. 3 | 1 |
|  | Hoverfly | Syrphidae | sp. 4 | 1 |
|  | Hoverfly | Syrphidae | sp. 5 | 1 |
|  | Non-syrphid Brachycera | Lauxaniidae | Unknown, not captured | 1 |
|  | Non-syrphid Brachycera | Muscidae | Unknown, not captured | 1 |
|  | Non-syrphid Brachycera | Tachinidae | sp. 2 | 1 |
|  | Non-syrphid Brachycera | Tachinidae | Unknown, not captured | 1 |
|  | Non-syrphid Brachycera | Unknown | not captured | 1 |
|  | Coleoptera | Cerambycidae | *Pidonia puziloi* | 2 |
|  | Bee | Apidae | *Bombus diversus* | 2 |
|  | Bee | Apidae | *Bombus honshuensis* | 5 |
|  | Bee | Apidae | *Bombus hypocrita*, not captured | 11 |
|  | Other Hymenoptera | Crabronidae | sp. | 1 |
|  | Lepidoptera | Nymphalidae | Argynnis paphia | 1 |
|  | Lepidoptera | Thyrididae | sp. | 1 |
